# HGCA2.0: An RNA-Seq Based Webtool for Gene Coexpression Analysis in *Homo sapiens*

**DOI:** 10.3390/cells12030388

**Published:** 2023-01-21

**Authors:** Vasileios L. Zogopoulos, Apostolos Malatras, Konstantinos Kyriakidis, Chrysanthi Charalampous, Evanthia A. Makrygianni, Stéphanie Duguez, Marianna A. Koutsi, Marialena Pouliou, Christos Vasileiou, William J. Duddy, Marios Agelopoulos, George P. Chrousos, Vassiliki A. Iconomidou, Ioannis Michalopoulos

**Affiliations:** 1 Centre of Systems Biology, Biomedical Research Foundation, Academy of Athens, 11527 Athens, Greece; 2 Section of Cell Biology and Biophysics, Department of Biology, National and Kapodistrian University of Athens, 15701 Athens, Greece; 3 Biobank.cy Center of Excellence in Biobanking and Biomedical Research, University of Cyprus, 2029 Nicosia, Cyprus; 4 School of Pharmacy, Aristotle University of Thessaloniki, 54124 Thessaloniki, Greece; 5 Centre of Basic Research, Biomedical Research Foundation, Academy of Athens, 11527 Athens, Greece; 6 University Research Institute of Maternal and Child Health and Precision Medicine, National and Kapodistrian University of Athens, 11527 Athens, Greece; 7 Personalised Medicine Centre, School of Medicine, Ulster University, Derry-Londonderry BT47 6SB, UK; 8 Engineering Design and Computing Laboratory, ETH Zurich, 8092 Zurich, Switzerland

**Keywords:** gene coexpression analysis, gene coexpression network, co-expression, RNA-Seq, transcriptomics, bioinformatics, webtool

## Abstract

Genes with similar expression patterns in a set of diverse samples may be considered coexpressed. Human Gene Coexpression Analysis 2.0 (HGCA2.0) is a webtool which studies the global coexpression landscape of human genes. The website is based on the hierarchical clustering of 55,431 *Homo sapiens* genes based on a large-scale coexpression analysis of 3500 GTEx bulk RNA-Seq samples of healthy individuals, which were selected as the best representative samples of each tissue type. HGCA2.0 presents subclades of coexpressed genes to a gene of interest, and performs various built-in gene term enrichment analyses on the coexpressed genes, including gene ontologies, biological pathways, protein families, and diseases, while also being unique in revealing enriched transcription factors driving coexpression. HGCA2.0 has been successful in identifying not only genes with ubiquitous expression patterns, but also tissue-specific genes. Benchmarking showed that HGCA2.0 belongs to the top performing coexpression webtools, as shown by STRING analysis. HGCA2.0 creates working hypotheses for the discovery of gene partners or common biological processes that can be experimentally validated. It offers a simple and intuitive website design and user interface, as well as an API endpoint.

## 1. Introduction

Genes that exhibit similar expression profiles in a large number of samples of different biological conditions, are considered coexpressed and tend to participate in similar biological processes or common metabolic pathways. The coexpression of genes, revealed through computational methods, can determine functional gene partners and, consequently, may be used to make assumptions about common pathway participation of a group of coexpressed genes, or to assign roles to genes of unknown function by consulting the known biological roles of the genes they are coexpressed with [1].

Gene coexpression analysis is performed on a specific organism, and uses samples from the same transcriptomic platform, that are processed in the same manner [2]. RNA-Seq [3] transcriptomic technology can massively study all transcripts of a tissue and has become the norm in gene expression estimation. As a consequence, RNA-Seq raw data constitute the main source for gene coexpression analysis. Depending on the type of samples used, coexpression analysis can be classified into two approaches: “condition-independent”, where primary data derive from healthy samples from a variety of tissues of an organism, and “condition-dependent”, where the samples come from a specific tissue or experimental condition [4]. RNA-Seq samples are procured either through in-house experiments or downloaded from public repositories, such as Gene Expression Omnibus (GEO) [5], Sequence Read Archive (SRA) [6], ArrayExpress [7], Expression Atlas [8], European Nucleotide Archive (ENA) [9], The Cancer Genome Atlas (TCGA) [10], and Genotype-Tissue Expression (GTEx) [11]. Public transcriptomic databases offer an ever-increasing amount of primary datasets which, if used for coexpression analysis, can produce findings that transcend the scope of each original experiment [12].

There are multiple RNA-Seq-based coexpression webtools for a vast variety of species [13,14,15,16], with a significant number of tools studying gene coexpression in *Homo sapiens* [17,18,19], a field of particular interest, as unravelling the coexpression relationships in human genes can lead to a better understanding of specific metabolic pathways that can potentially elucidate the primary molecular mechanisms behind diseases [20] or reveal novel gene functional partners [4,21]. Here, we describe HGCA2.0, a web-based coexpression tool for *Homo sapiens*, created using 3500 representative bulk RNA-Seq samples from GTEx, and we present several use cases for human genes.

## 2. Materials and Methods

### 2.1. Transcriptomic Data Processing

GTEx version 8.0 RNA-Seq gene read count and TPM-normalised [22,23] expression data (phs000424.v8.p2, 5 May 2017 released), as well as their corresponding metadata, were downloaded from GTEx Portal [11], which offers high-quality curated RNA-Seq data from various human tissues, processed with the same protocol, and ArrayExpress. This GTEx version includes RNA-Seq data from 17,382 samples of 54 tissues from 948 post-mortem donors [24]. GTEx TPM expression data for 56,200 genes were only used to discover non-expressed genes. The lowest non-zero TPM expression value replaced zero TPM values, so that all expressions could be log_2_ transformed. Genes with zero standard deviation across all samples were identified and subsequently removed, this accounted for 322 genes. All 44 Y chromosome pseudoautosomal genes, denoted by a “_PAR_Y” suffix in their Ensembl [25] gene version code, were among them. Furthermore, another 447 genes with deprecated Ensembl gene stable IDs according to Ensembl release 99 Biomart [26], were also removed. Finally, cell-line samples were deleted, leaving 16,704 samples remaining. Afterwards, read count data corresponding to the remaining genes and samples were normalised using the normalizeTissueAware function in YARN [27], a wrapper for the qsmooth normalisation algorithm [28], which normalises all samples with the assumption that the statistical distribution of counts should be similar among samples of the same tissue rather than across all samples.

### 2.2. Gene Coexpression Tree Construction

Through custom PHP scripts, pairwise correlations between samples or genes were calculated using Pearson’s Correlation Coefficient (PCC or *r*-value) [29]. The correlation values were transformed to distance values using the *d* = 1 − *r* formula [30], and hierarchical clustering of samples or genes was performed on the transformed pairwise distance values through the Phangorn [31] R package implementation of UPGMA [32].

In order to determine the representative GTEx samples, and create the HGCA2.0 gene coexpression tree based on them, we followed a procedure previously described [33]: using the normalised expression data of 55,431 *Homo sapiens* genes in 16,704 samples, *r*-values were calculated for each sample pair and a sample distance matrix was created in PHYLIP format [34]. This ensures that all distances have positive values, with a range between 0 and 2, where 0 represents complete correlation, and 2 represents complete anti-correlation. A sample correlation tree in Newick format [35] was produced, using the distance matrix of samples as input. Each leaf of the produced tree corresponded to a unique GTEx sample.

As our goal was to study the global (i.e., condition/tissue-independent) coexpression landscape of *Homo sapiens*, the most representative samples of the dataset were selected to minimise tissue bias (Figure S1). Tree pruning was performed on the tree of 16,704 sample-leaves using a previously described custom-made iterative PHP algorithm [2], which automatically prunes adjacent leaves in a cladogram, producing a tree of 3500 leaves which corresponded to the most distinct representative samples (Figure S2).

To calculate the *r*-values between all gene pairs, gene expression values of those 3500 samples were used to create a distance matrix of genes, in a similar manner as the distance matrix of samples. The gene distance matrix was used to construct a coexpression tree of 55,431 genes, which were represented as leaves. Genes lying in the same clade are considered coexpressed.

### 2.3. External Data Collection and Biological Term Enrichment Calculation

HGNC [36] gene symbols and descriptions, as well as Ensembl gene stable IDs, were downloaded from Ensembl Biomart, gene ontologies from Gene Ontology [37], biological pathways from KEGG [38] and WikiPathways [39], transcription factor target genes from ENCODE [40] through Harmonizome [41] and ReMap2020 [42], gene-disease associations from OMIM [43] and DisGeNet [44], protein domains from Pfam [45], and cytogenetic band coordinates from the NCBI Genome Decoration Page (https://www.ncbi.nlm.nih.gov/genome/tools/gdp) (accessed on 20 January 2023). All data were downloaded and parsed using in-house PHP scripts, either through the BioMart XML-based data retrieval system or directly from their respective websites. We intend to update the biological term data each time a new GTEx version is released and a new gene coexpression tree is calculated. Gene term enrichment analysis *p*-value calculations are based on the Hypergeometric Distribution [46] and the False Discovery Rate (FDR) [47] corrected.

### 2.4. Webtool Implementation and Usage

A MySQL relational database was designed to store all required data, i.e., gene expression values and metadata for each sample, as well as human gene biological terms. The web server is hosted on a 16-core, 64 GB memory, Ubuntu 22.04 Linux system. Website development was based on fully validated HTML5 by HTML validator (https://www.gueury.com/) (accessed on 20 January 2023) and CSS, along with Bootstrap (https://getbootstrap.com/) (accessed on 20 January 2023) styling library and JavaScript. All backend scripts were written in PHP and run on an Apache 2.4.52 web server with verified HTTPS protocol.

**Figure 1:**
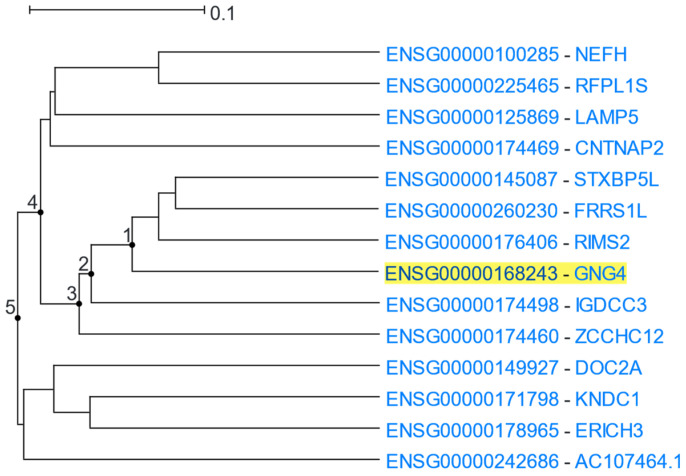
The five branching points (depicted as numbered dots), from the driver gene leaf (*GNG4*) until the root of the clade, constitute the internal nodes of this coexpression clade.

On the main page of the website, the user initially inputs a human Ensembl gene stable ID or HGNC gene symbol (henceforth, the “driver gene”), and a gene coexpression clade whose size is closest to 25 genes is displayed, along with a scale bar (referring to the distance-transformed PCC) at the top. Genes in the coexpression clade are represented as terminal nodes (“leaves”), which are progressively connected through internal nodes, which, in turn, are represented as branching points (Figure 1). The internal node number, from the driver gene leaf to the root of the clade, is displayed below the tree visualisation. The clade size can be increased or decreased by adding or subtracting internal nodes, with a maximum clade size of up to 25% of the genes studied. Each clade leaf name contains an Ensembl gene stable ID, an HGNC gene symbol, and a gene description. To change driver gene, the user clicks on a different Ensembl gene stable ID. Although the same coexpression clade will be displayed, when choosing a driver gene located in a different subclade, this subclade can be isolated and studied by reducing the clade size. To find more information on any gene of the clade, the user may click on a gene symbol to visit the corresponding gene entry in Genecards [48]. The constructed coexpression clade can be downloaded in Newick format [35] or viewed in iTOL tree viewer [49]. A table located below the gene clade, contains the descriptions of the gene-leaves. The gene list of the clade can also be downloaded or exported to the g:Profiler [50], Genemania [51], Pathway Commons [52], FLAME [53], STRING [54], and EnrichR [55] websites for further analyses.

To perform a gene term enrichment analysis using the genes of the current clade, an enrichment category needs to be selected from a drop-down menu, which appears right below the coexpression clade. The analysis is performed on the fly and the enrichment results are displayed on the enrichment summary table. Only terms with an FDR-adjusted *p*-value ≤ 0.05 are presented, and ranked in *p*-value ascending order. Furthermore, for each term, its over-representation rate (observed/expected) and hit percentage (appearance in the coexpression clade/appearances in all available genes) are displayed. The change in clade size affects the results of the enrichment analysis. A smaller clade might not be able to deem terms as statistically significant, and only on a tree of increased size may those terms be revealed. On the other hand, a larger clade might contain smaller subclades of genes with different biological functions which might be revealed by decreasing the tree size. As such, it is at the user’s discretion to determine the optimal tree size, through the observation of the fluctuations of the FDR-adjusted *p*-values of the enriched biological terms or other metrics. Below the enrichment table, a full list of the genes of the clade, along with all the terms of that category that describe them, is displayed, linking to the corresponding website entries.

### 2.5. API Access

HGCA2.0 provides access to all coexpression and enrichment results through a public JSON-based Application Programming Interface (API) endpoint, keyed on an Ensembl gene stable ID, a tree node number and, optionally, an enrichment analysis category 2-character keyword. For example, using https://www.michalopoulos.net/hgca2.0/api/ENSG00000114391/5/bp (accessed on 20 January 2023) returns the coexpression clade of the driver gene ENSG00000114391 with 5 internal nodes in Newick format, the driver gene details, the coexpression clade gene list, and the enriched “Gene Ontology: Biological Process” terms ranked by *p*-value. If a wrong (or no) keyword is typed, then no enrichment analysis will be performed. Instructions to developers can be found in the API section of the “Help” page of the HGCA2.0 website.

### 2.6. STRING Analysis

STRING is a webtool performing protein-protein interaction (PPI) network construction by incorporating known and predicted interactions between proteins, as well as interactions based on text-mining, co-expression, and homology, which are scored relative to their evidence strength. Additionally, STRING offers multiple network metrics and built-in enrichment analysis categories. To perform benchmarking of HGCA2.0, and 4 other popular webtools also studying condition-independent gene coexpression analysis in *Homo sapiens*, i.e., COXPRESdb [56], GeneFriends [18], ARCHS^4^ [57], and SEEK [58], various metrics of STRING v11.5 were used as independent comparison measures: The gene of interest was used as the driver gene in HGCA2.0, to produce a default coexpression clade (a coexpression clade with ~25 leaves) and its corresponding list of coexpressed genes. The same driver gene was used as the input for the rest of the coexpression webtools as follows: In COXPRESdb, hsa-u.4 was used as the coexpression collection for analysis, in GeneFriends, both GTEx and SRA were selected as data source with samples of all tissues, in SEEK, multi-tissue profiling dataset was selected and ARCHS^4^ was used as is, since there were no configurations available. The gene lists of the top-ranked coexpressed genes (including the driver gene), as well as that of HGCA2.0, underwent STRING multiple protein analysis, ensuring that each list contained the same number of genes mapped by STRING. STRING protein-protein interaction network generation was performed by removing any “co-expression” interactions and setting a high confidence cut-off (0.700). The metrics used for the evaluation of the tools include, “Number of Edges”, “Expected Number of Edges”, “Average Node Degree”, “Avg. Local Clustering Coefficient” [59], “PPI enrichment *p*-value”, and Gene Ontology: Biological Process and KEGG Pathways biological term enrichment analyses available in STRING.

## 3. Results

### 3.1. Use Cases

#### 3.1.1. Ribosomal Proteins

**Figure 2:**
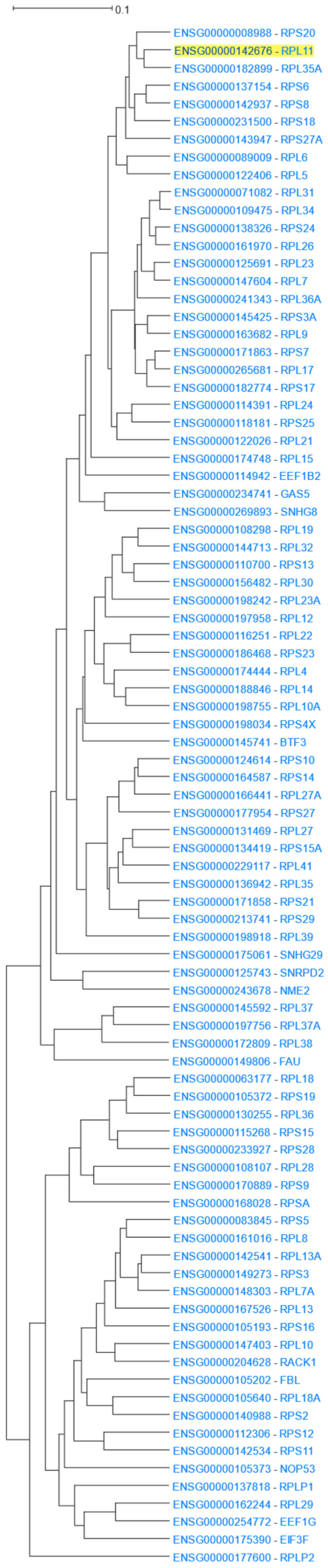
*RPL11* 14 internal node HGCA2.0 coexpression clade.

The human 80S ribosome is a complex of 80 proteins and 4 RNA molecules [60]. *RPL11*, coding Ribosomal Protein L11, was used as the input to HGCA2.0. The webtool produced a clade that was expanded up to 14 internal nodes and contained 87 genes. Of those genes, 75 were ribosomal proteins (Figure 2). Enrichment analyses in all Gene Ontology aspects, as well as in KEGG and WikiPathways, highlighted ribosome-related terms, achieving very low *p*-values (Table 1). In addition, Pfam showed several ribosome-specific families of proteins, and DisGeNet linked genes of the clade to Diamond-Blackfan anaemia, a known ribosomopathy [61], and to several of the disease’s clinical features, such as short stature, cleft palate, and thumb deformities [62]. Finally, ENCODE revealed a large number of enriched transcription factors targeting almost all of the 87 coexpressed genes.

#### 3.1.2. Metallothioneins

**Figure 3:**
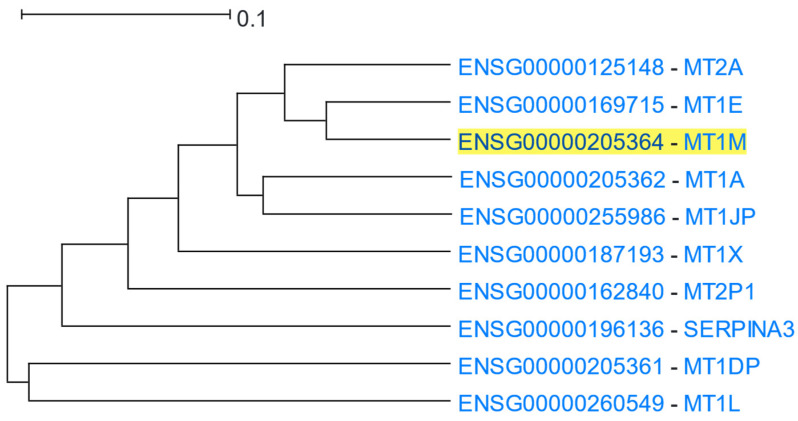
HGCA2.0 *MT1M* 5 internal node coexpression clade.

Metallothioneins have a high percentage of cysteine residues and bind to various heavy metals. They are regulated at the transcriptional level by both heavy metals and glucocorticoids [63]. *MT1M* (Metallothionein 1M) was used as the driver gene in HGCA2.0. The clade produced was reduced to 7 internal nodes and contained 10 genes (Figure 3), 9 of which belonged to metallothioneins, and 4 of them being insufficiently annotated pseudogenes. A GO Biological Process enrichment analysis identified terms related to stress response to metal ions, such as copper, cadmium, and zinc, and detoxification of inorganic compounds, such as copper ions (Table 2). A GO Molecular Function analysis also proposed binding to metals, such as zinc ions, as enriched terms. A KEGG biological pathway analysis highlighted the term “mineral absorption” in humans, and WikiPathways displayed the terms “zinc homeostasis” and “copper homeostasis”. A Pfam analysis assigned the proteins of the coexpressed genes to the Metallothionein family. Finally, a transcription factor analysis via ReMap revealed two transcription factors of the zinc finger family (zinc finger proteins 879 and 26) as targeting the genes of the clade.

#### 3.1.3. MHC Class I and Class II Protein Clusters

**Figure 4:**
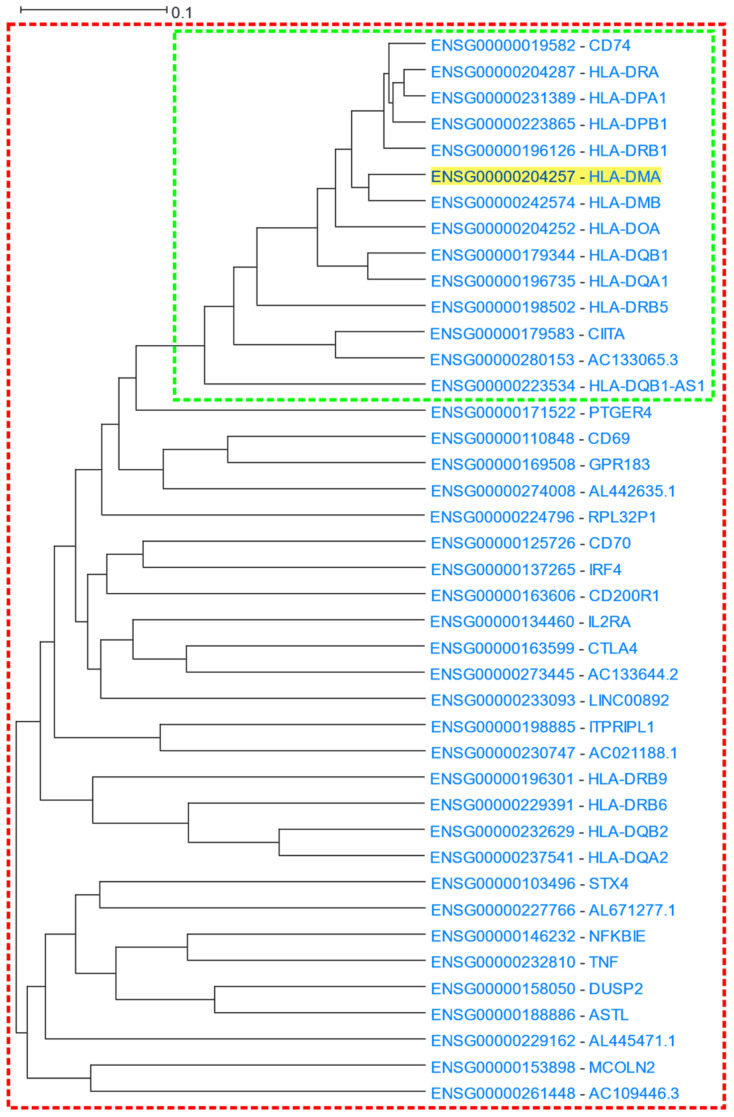
HGCA2.0 *HLA-DMA* coexpression clade. The 7 internal node clade is included in the green box, while the expanded 14 internal node clade is included in the red box.

Major Histocompatibility Complex class II (MHC-II) proteins are known to function at the early stages of immune response, by presenting processed peptides to CD4+ T-lymphocytes [64]. HLA-DM is a MHC-II protein heterodimer consisting of an α and a β chain which are encoded by the *HLA-DMA* and *HLA-DMB* genes, respectively [65]. HLA-DM regulates the loading of peptides into MHC-II molecules of the antigen-presenting cells [66,67]. *HLA-DMA* (Major Histocompatibility Complex, Class II, DM Alpha) was used as a driver gene in a HGCA2.0 analysis. A clade that was reduced to 7 internal nodes was produced. The clade contained 14 gene-leaves, 13 of which were HLA or HLA-related genes (Figure 4). The most correlated gene to *HLA-DMA* was that of its binding partner, *HLA-DMB* (Major Histocompatibility Complex, Class II, DM Beta). A GO enrichment analysis revealed terms of antigen presentation via MHC class II in Biological Process aspect, binding to MHC class II proteins in Molecular Function aspect, and MHC class II complex in Cellular Component aspect (Table 3). A KEGG biological pathway analysis showed the presentation and processing of antigens in humans as the top function, and a Pfam analysis showed as over-represented, families of α and β chains of MHC class II and a protein family corresponding to the C1-set domain of immunoglobulin. A ReMap enrichment analysis showed over 40 enriched transcription factors, with the top two being SMAD5 and ZBED1. In the coexpression clade, the only gene which was not described by any biological term was a “To be Experimentally Confirmed” (TEC) non-coding gene, AC133065.3, whose most correlated gene was *CIITA*. Given that the genomic coordinates of AC133065.3 fall within *CIITA* genomic boundaries, both have the same transcription orientation (Figure S3) and display similar expression patterns, AC133065.3 might constitute a *CIITA* alternative monoexonic transcript, sharing common transcriptional regulatory mechanisms.

The coexpression clade was further expanded up to 14 internal nodes, revealing a total of 41 genes (Figure 4), among which additional MHC Class II family genes (*HLA-DQB2*, *HLA-DQA2*, *HLA-DRB9* and *HLA-DRB6*) were identified. Moreover, essential genes related to innate and adaptive immune response (e.g., *TNF*, *NFKBIE*, *IRF4*, *IL2RA*, *STX4*) were also identified. In particular, *TNF* (Tumour Necrosis Factor) encodes for a pleiotropic cytokine, which binds to its membrane receptors, TNF receptor type I (TNFR1) and TNF receptor type II (TNFR2), and participates in cellular responses [68,69]. *NFΚBIE* encodes for an essential negative feedback regulator of the NF-κB transcription factor which regulates immune responses, B cell proliferation and survival, cancer phenotype establishment, etc., [70,71,72]. In addition, the IRF4 transcription factor, a member of the IRF family, has a regulatory role in the immune response, proliferation, and differentiation of immune system cells [73,74]. An enrichment analysis on the expanded coexpression clade, revealed that terms related to defence and immune response were more prominent compared to the analysis for the initial 7 internal node clade: “adaptive immune response” had an adjusted *p*-value of 6.2 × 10^−17^ in the 14 internal node clade compared to 1.4 × 10^−11^ in the 7 internal node one.

**Figure 5:**
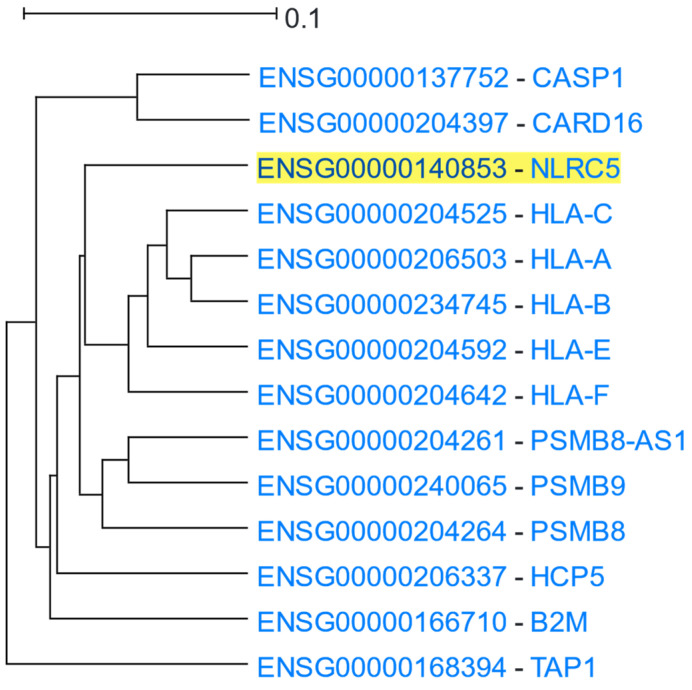
HGCA2.0 *NLRC5* 6 internal node coexpression clade.

NLR family CARD domain containing 5 (NLRC5) is constitutively expressed in a multitude of human tissues, but predominantly in hematopoietic cells. NLRC5 contains a nuclear localisation signal (NLS) enabling its translocation into the nucleus upon induction of cells by certain stimuli. NLRC5 lacks a DNA-binding domain and interacts with a plethora of transcription factors and multi-protein complexes to exert its well-described regulatory role in stimulus-induced activation of Major Histocompatibility Complex class I (MHC-I) genes [75,76]. An *NLRC5*-centered HGCA2.0 analysis generated a clade that was reduced to 6 internal nodes, which contained 14 genes in total (Figure 5). The closest neighbouring leaves were composed of MHC-I members (*HLA-A/B/C/E/F*), in line with their aforementioned NLRC5-induced transactivation. Importantly, *B2M*, *PSMB9*, and *TAP1* [75,76] were composites of the second mostly correlated subclade. A GO enrichment analysis underscored the antigen processing and presentation via MHC-I molecules as one of the most significantly over-represented terms (Table 4). Furthermore, NLRC5 has been proposed as a main component of NLRP3 inflammasome reconstitution, in response to immunogenic stimuli or Damage Associated Molecular Patterns (DAMPs). Inflammasome’s activity, among other things, mediates Caspase 1 (CASP1) maturation [77]. Both *CASP1* and its inhibitor, *CARD16*, were identified as significantly coexpressed, supported by GO Biological Process, KEGG pathways, and WikiPathways analyses, which highlighted immune-related inflammatory responses and cytokine-mediated signalling pathways as enriched. The inflammasome complex along with the MHC-I complex were also identified as the most enriched GO Cellular Component terms associated with *NLRC5* and its coexpressed genes.

#### 3.1.4. STAT1 Transcription Factor

**Figure 6:**
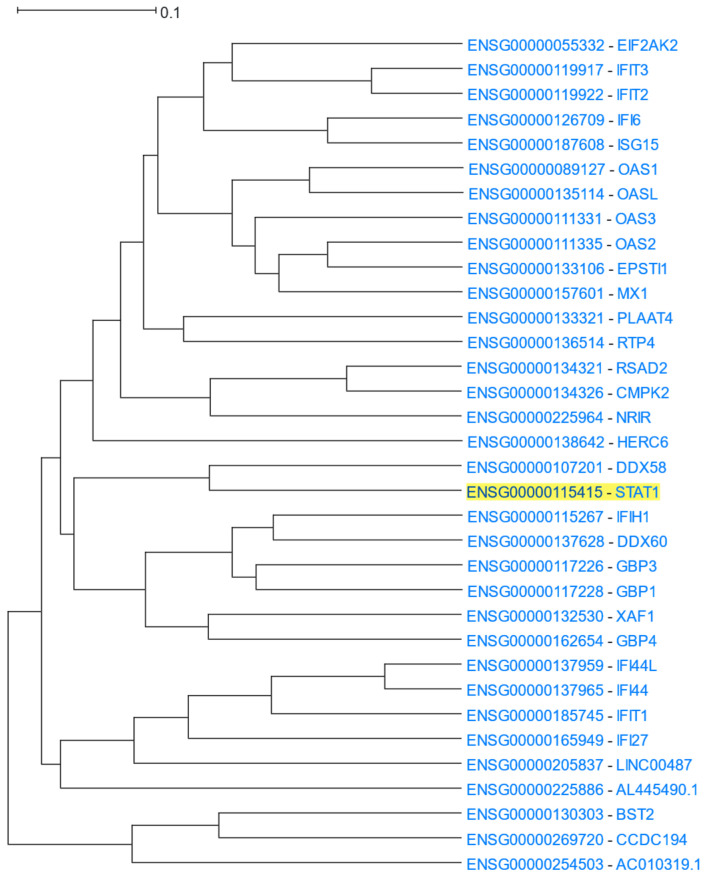
HGCA2.0 *STAT1* 5 internal node coexpression clade.

STAT1 (Signal Transducer and Activator of Transcription 1) is a transcription factor and a member of the STAT family of proteins. STAT1 is activated by type I interferons, mediates the expression of various genes that play a role in cell survival in response to various stimuli and pathogens, and can form dimers with other members of the same family [78]. *STAT1* was used as the driver gene to HGCA2.0 and the produced clade was expanded to 5 internal nodes with 34 gene-leaves, many of which were related to interferons (Figure 6). A GO Biological Process enrichment analysis highlighted defence response to virus terms as the top enriched ones (Table 5). KEGG and DisGeNet over-representation analyses showed an association with various viral diseases. A WikiPathways enrichment analysis showed the involvement of *STAT1* and other clade genes, such as genes belonging to the OAS family (*OAS1*, *OAS2*, *OAS3*, *OASL*), in the response to human coronaviruses. Finally, both ENCODE and ReMap transcription factor analyses showed STAT2 as the top transcription factor, targeting more than 2/3 of the genes of the clade.

#### 3.1.5. TMPRSS2 in Relation to COVID-19 Infection

**Figure 7:**
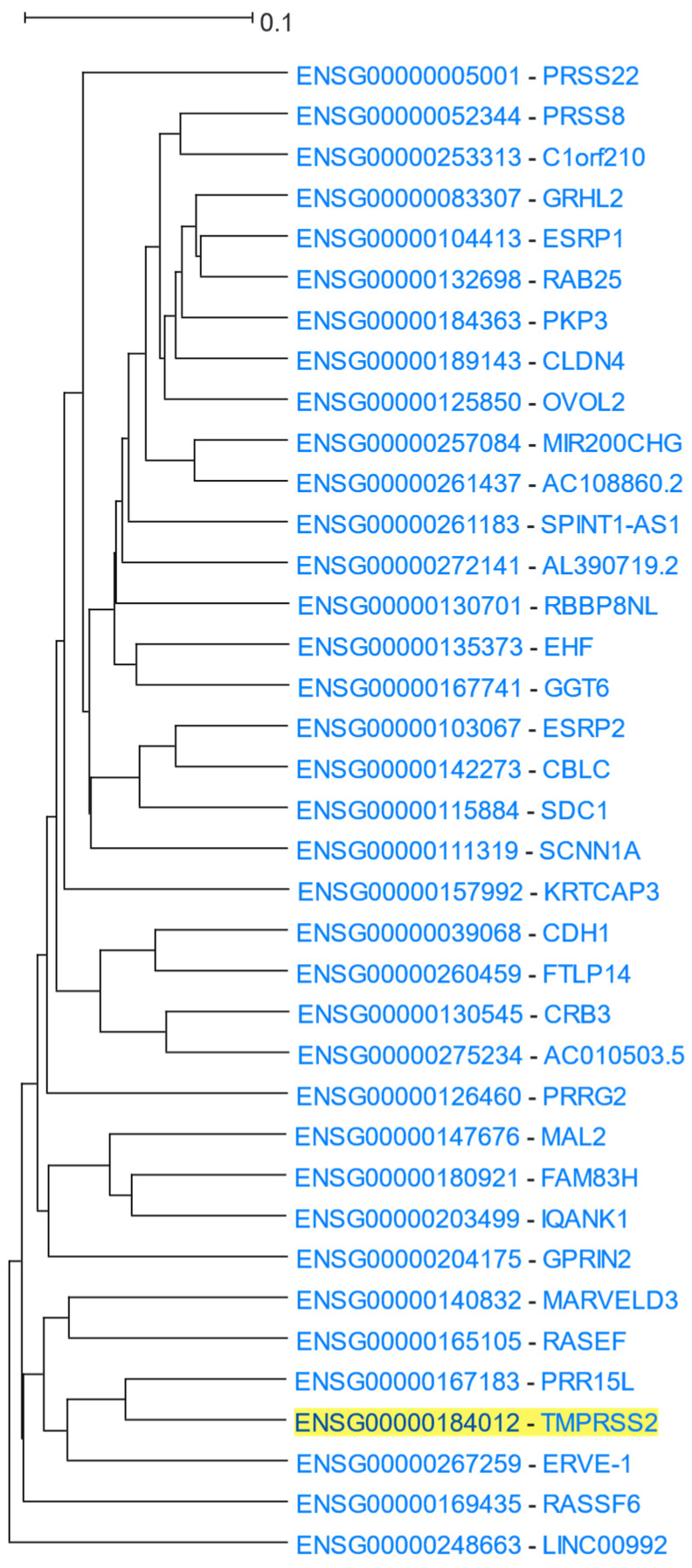
HGCA2.0 *TMPRSS2* 6 internal node coexpression clade.

*TMPRSS2* (Transmembrane Serine Protease 2) encodes for a transmembrane protein belonging to the type 2 serine protease family with a role in epithelial homeostasis. Several viruses use TMPRSS2 for cell invasion [79]. The SARS-CoV-2 virus has been found to infect the human body via the ACE2 receptor in combination with TMPRSS2 [80]. *TMPRSS2* was used as the driver gene in a HGCA2.0 analysis and the produced clade was expanded up to 6 internal nodes and contained 37 genes (Figure 7). A GO Biological Process analysis revealed terms related to epithelial cells and intercellular binding, which is in agreement with SARS-CoV-2 attachment to epithelial cells (Table 6). GO Cellular Component and KEGG biological pathways analyses also showed terms related to intercellular binding. Among the top three transcription factors discovered by ENCODE, two were factors related to the zinc finger family and the third one was ESR1 (Estrogen Receptor 1). Additionally, a ReMap analysis found, among several other transcription factors, that ESR1 targets 36 out of all 37 genes of the coexpression clade. The presence of ESR1 as a factor targeting *TMPRSS2* and genes which are coexpressed with it, may explain the distinct fatality patterns between males and females [81,82].

#### 3.1.6. Late Cornified Envelope Genes

**Figure 8:**
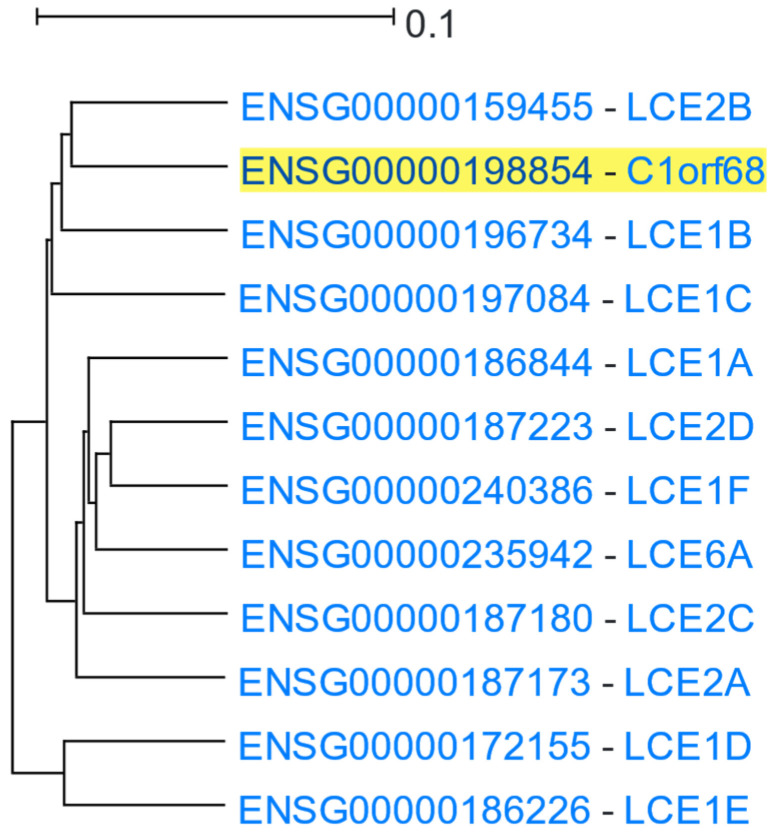
HGCA2.0 *C1orf68* 5 internal node coexpression clade.

**Figure 9:**
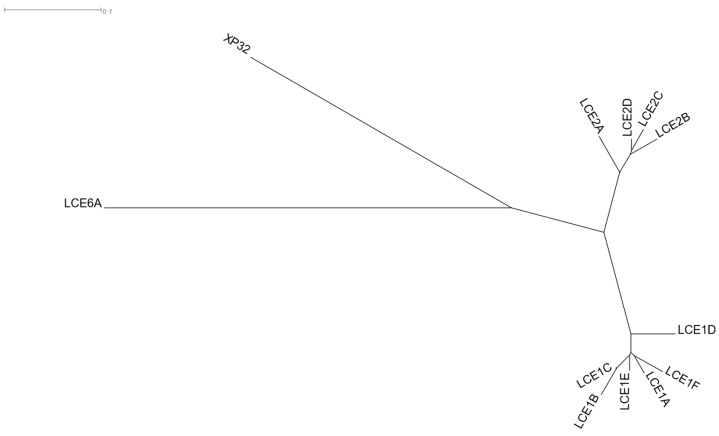
Phylogenetic tree resulting from MUSCLE multiple sequence alignment of the protein sequences of the genes of the HGCA2.0 *C1orf68* (XP32) coexpression clade, as viewed by Dendroscope [91].

Late cornified envelope (LCE) clusters of genes are stratum corneum proteins responsible for keratinisation. They are located in a ~380 Kbps region of 1q21.3 cytoband (Figure S4), which is part of a wider genomic region stretching ~1.9 Mbps, known as the epidermal differentiation complex [83]. LCE 1 and 2 group genes are primarily expressed in the skin [84]. *C1orf68* (Chromosome 1 Open Reading Frame 68), also known as Skin-Specific Protein 32 (XP32), is located in the genomic region between the LCE 1 and 2 clusters. *C1orf68* was used as the driver gene in a HGCA2.0 analysis, and the resulting clade was reduced to 5 internal nodes containing 12 genes, all of which, except for *C1orf68*, were LCE genes (Figure 8). A GO Biological Process enrichment analysis showed “keratinization” and “epidermis development” as top terms (Table 7), and WikiPathways revealed the “Vitamin D Receptor Pathway” term as over-represented. Pfam classified 10 of the coexpressed genes into the LCE protein family, and chromosome band analysis indicated all genes as located in 1q21.3, suggesting that this genomic co-localisation may be responsible for coexpression. A multiple protein sequence alignment of the coexpressed genes (Figure S5), using MUSCLE [85], showed a high degree of similarity between LCE1 and LCE2 genes, with the genes of each family being clustered in distinct subclades (Figure 9). The topology of the phylogenetic tree indicates that *C1orf68* and *LCE6A* are ancient paralogues of the LCE 1 and 2 gene groups. SignalP 6.0 [86] predicted that none of the proteins contained any signal peptide. As none of the proteins of the coexpressed genes had any solved structure, a model could not be constructed in SWISS-MODEL [87] to predict the C1orf68 structure through homology modelling. The AlphaFold [88] prediction for C1orf68 (UniProt ID: Q5T750) was a Beta structure which matches with the 2-solenoid architecture (CATH ID: 2.150) of CATH [89]. On the other hand, AlphaFold predicted serpentine protein structures for the LCE proteins (e.g., UniProt ID: Q5T7P2 for LCE1A). The discovery of several tandem repeats (Figure S6) in the C1orf68 protein sequence by HHrepID [90] may justify the 2-solenoid architecture prediction.

#### 3.1.7. Heat Shock Protein 90

Heat shock proteins (HSP) were named after their elevated expression during heat shock response [92]. The HSP90 (90kDa) chaperone machinery plays an important role in the regulation of proteostasis during physiological and stress conditions in eukaryotic cells, and it is involved in many cellular processes, beyond protein folding and assembly, such as signal transduction, cell cycle control, DNA repair, development, immune response, and neurodegenerative diseases [93]. HSP90 has three structural domains: the N-terminal domain (NTD), in which the ATP binding site is located, the middle domain (MD), and the C-terminal domain (CTD) which is responsible for the dimerisation [94].

There are two HSP90 genes which encode HSP90A and HSP90B. *HSP90A* is induced by heat shock. It appears across cytosol in all eukaryotes and is duplicated in vertebrates into *HSP90AA1* and *HSP90AB1* [95]. *HSP90B1* is constitutively expressed in the cytosol [96]. It is present in the endoplasmic reticulum in all eukaryotes, with the exception of some fungal species, and is associated with molecular chaperones which transmit information within the compartment and help transport “passenger proteins” across membranes.

**Figure 10:**
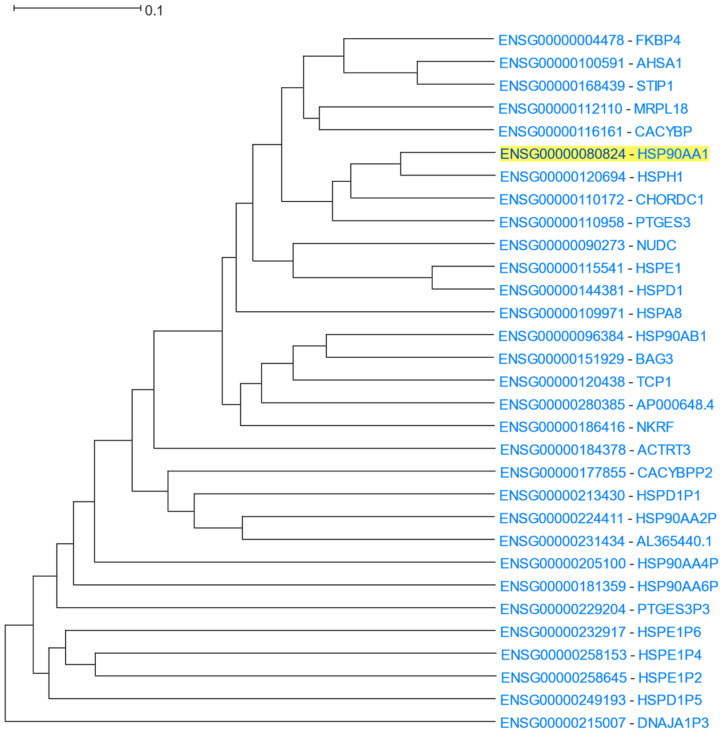
HGCA2.0 *HSP90AA1* 14 internal node coexpression clade.

Using *HSP90AA1* as the driver gene, HGCA2.0 produced a coexpression clade which was expanded up to 14 internal nodes and contained 31 gene-leaves, 16 of which were HSP or HSP-related genes (Figure 10). *HSPH1* (Heat shock protein family H) and *CHORDC1* (cysteine- and histidine-rich domain-containing protein) appear as the most highly coexpressed genes with *HPS90AA1*. Indeed, *HSP90AA1* is highly coexpressed with *HSPH1* during head and neck squamous cell carcinoma (HNSCC), which means that these factors could be either prognostic biomarkers or potential clinical targets [97]. Furthermore, HSP90 complexes interact with CHORDC1 as an ADP-dependent HSP90-interacting protein [98]. The *HSP90AA1* paralog, *HSP90AB1*, is also found on a neighbouring subclade. A GO Biological Process enrichment analysis displayed “protein folding”, “regulation of cellular response to heat”, and “chaperone-mediated protein folding”, as top processes, the GO Molecular Function showed “unfolded protein binding”, “chaperone binding”, and “heat shock protein binding”, and GO Cellular Component analysis revealed “chaperone complex” as the top term (Table 8). An ENCODE analysis exhibited HSF1 (heat shock transcription factor 1) and PPARGC1A (PPARG coactivator 1 alpha) as the top transcription factors related to *HSP90AA1*. This association between HSF1 and *HSP90AA1* is confirmed by studies that suggest that HSF1, the master transcriptional regulator of heat shock response, allows the inducible expression of *HSP90AA1* upon binding to heat shock elements (HSEs), which are located upstream of *HSP90A* [96]. A DisGeNET analysis showed Tauopathies as one of the top related diseases associated with *HSP90AA1*. This finding is in accordance with previous studies, which proposed that changes in the expression levels of HSP90s and their co-regulators could drive tau deposition and neurotoxicity, leading to Alzheimer’s disease (AD) and other neurodegenerative diseases (tauopathies) [99]. Finally, a Pfam analysis displayed “CS domain”, “HSP90”, and “HSP70” as over-represented families which are related to *HSP90AA1*.

**Figure 11:**
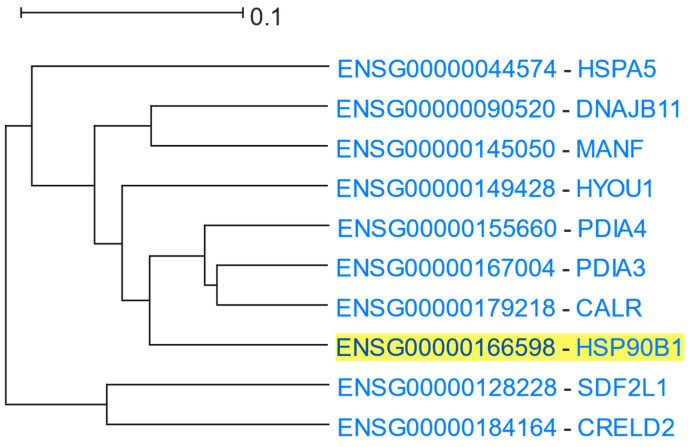
HGCA2.0 *HSP90B1* 5 internal node coexpression clade.

Using *HSP90B1* as the driver gene, HGCA2.0 produced a clade that was reduced to 5 internal nodes having 10 gene-leaves, 3 of which were HSP or HSP-related genes (Figure 11). A GO Biological Process enrichment analysis displayed “response to endoplasmic reticulum stress”, “response to topologically incorrect protein”, and “protein folding in endoplasmic reticulum”, as the top processes, the GO Molecular Function showed “protein disulfide isomerase activity”, “intramolecular oxidoreductase activity (transposing S-S bonds)”, “chaperone binding”, and “unfolded protein binding”, and a GO Cellular Component analysis revealed “endoplasmic reticulum lumen” and “endoplasmic reticulum chaperone complex” as the top terms (Table 9). An ENCODE analysis exhibited SP2 (Sp2 transcription factor), as the top functional element related to *HSP90B1*, whereas DisGeNET showed Spinocerebellar Ataxia 17 as one of the top related diseases associated with it.

“Chaperone binding” and “unfolded protein binding” are shared enriched terms in both coexpression clades, as heat shock proteins interact with unfolded proteins preventing or reversing their aggregation, assisting their refolding to native structure [100]. “Regulation of cellular response to heat” appears only in the first clade because *HSP90AA1* and *HSP90AB1* are stress-induced while *HSP90B1* is constitutively expressed [96]. That difference in expression patterns also explains why *HSP90B1* is located in a different clade than that of *HSP90AA1* and *HSP90AB1*.

#### 3.1.8. Neurovascular Genes

**Figure 12:**
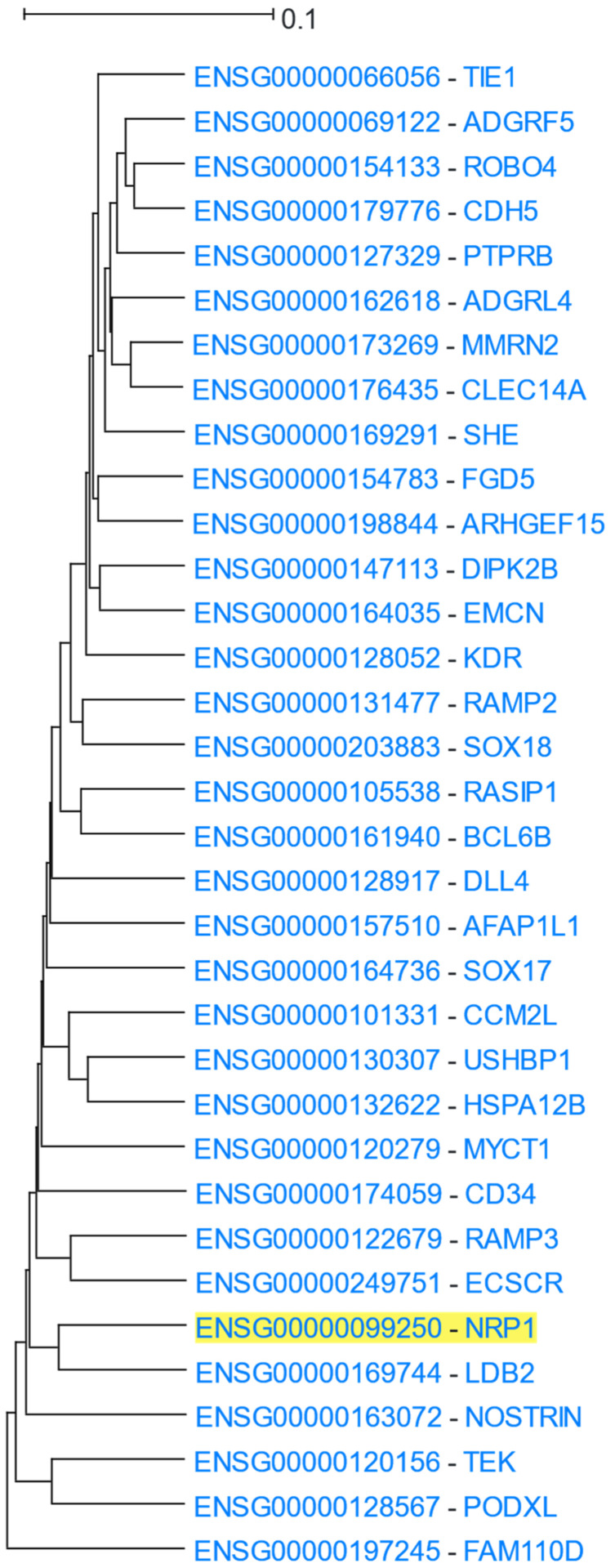
HGCA2.0 *NRP1* 5 internal node coexpression clade.

*NRP1* (Neuropilin 1) is a receptor for vascular endothelial growth factor (VEGF) and a member of the semaphorin family of proteins. It has been shown to regulate angiogenesis and vascular permeability [101]. A *NRP1* analysis in HGCA2.0 produced a coexpression clade that was expanded to 5 internal nodes with a total of 34 genes (Figure 12). A GO Biological Process enrichment analysis (Table 10) highlighted terms related to cardiovascular system development, a result supported by a DisGeNet analysis, which contained blood-vessel-related diseases and anomalies. In addition, a large number of enriched terms were related to NRP1′s role as a receptor for VEGF, such as “vascular endothelial growth factor-activated receptor activity” in the GO Molecular Function, and “Robo4 and VEGF Signaling Pathways Crosstalk” and “VEGFA-VEGFR2 Signaling Pathway” in WikiPathways.

#### 3.1.9. Olfactory Receptors

Olfactory receptors are a family of ~1000 genes responsible for the sense of smell, with about 60% constituting pseudogenes [102,103], and are all expressed in the olfactory sensory neurons [104]. Each odourant ligand can be recognised by multiple olfactory receptors with different affinity, and specific odourants can be bound to certain olfactory receptor families. The monogenic and monoallelic expression of olfactory receptors in a single olfactory neuron cell is due to the stochastic activation of a single allele of a single gene from an array of olfactory receptor genes [104]. *OR1D2* (Olfactory Receptor Family 1 Subfamily D Member 2) was used as the input to HGCA2.0 and the resulting clade was expanded to 98 internal nodes containing 398 genes (Figure S7), 220 of which were olfactory receptors. Among the olfactory receptor leaves, smaller subclades of other gene families, such as Interferon Alpha family or Pregnancy Specific Beta-1-Glycoprotein family, were encountered. A particular characteristic of that clade was that its internal nodes were very close to its root, i.e., the cophenetic distances [105] of all its coexpressed gene pairs were similar. Cophenetic distances refer to the pairwise distances between genes, as these are depicted on a gene coexpression tree [32]. Essentially, the coexpression tree represents a distance matrix, where pairwise distances between its genes correspond to their Cophenetic distances. The comparison of the original Pearson correlation-based distance matrix with the Cophenetic distance matrix derived from the UPGMA-constructed coexpression tree can be used to measure the quality of the hierarchical clustering. When the pairwise distances between all 839 olfactory receptor genes and pseudogenes studied in HGCA2.0 were examined, the average distance prior to clustering was ~0.93. Their respective cophenetic distances derived from the UPGMA tree were also examined, with the average distance being ~0.96. Those distances corresponded to ~0.07 and ~0.04 Pearson correlation coefficients, respectively, meaning that, in any case, there was almost no correlation between any olfactory gene pair. When a STRING analysis was performed, using all 839 olfactory receptor genes that HGCA2.0 studied, STRING recognised only 376 non-pseudogenes. Out of 70,500 olfactory receptor gene pairs, 2973 had Pearson correlation-based coexpression interaction scores ranging from 0.048 to 0.520, with only two of them exceeding the default 0.400 cut-off.

A HGCA2.0 enrichment analysis of GO Biological Process highlighted terms related to stimulus detection as over-represented (Table 11). “Detection of a chemical stimulus involved in sensory perception of smell” which describes 180 of the 398 genes of the clade, was a top term. For this specific term, there are 387 genes described by it in the gene background library, 180 of which (46.5%) are located in this coexpression clade. Likewise, the GO Molecular Function highlighted the terms “olfactory receptor activity” and “G protein-coupled receptor activity”, and a GO Cellular Component analysis showed the coexpressed genes as part of membranes. The aforementioned terms are in accordance with the fact that olfactory receptors are members of the large family of G-protein-binding receptors and are therefore naturally associated with the cell membrane. A KEGG pathways analysis similarly highlighted the term “olfactory transduction”, and a Pfam analysis classified the same 180 genes into the olfactory receptor family. In addition, a WikiPathways analysis showed top terms for G-protein coupled receptors and interferon-mediated signalling pathways, the latter being enriched due to the appearance of 4 IFNA-family genes in the coexpression clade.

In order to determine how the 839 olfactory receptor genes were distributed across the coexpression tree, a sliding window approach was implemented. The *OR1D2* coexpression clade of 398 genes, was discovered to be the largest one, containing 180 olfactory receptor genes and 40 olfactory receptor pseudogenes. Another distinct olfactory receptor clade of 135 genes, contained 41 olfactory receptor genes and 41 olfactory receptor pseudogenes. This clade can be displayed by using *OR51A7* as the driver gene and expanding the resulting coexpression clade to 77 internal nodes. The remaining 537 olfactory receptor genes studied in HGCA2.0 were scattered throughout the coexpression tree.

#### 3.1.10. Glucocorticoid Receptor Signalling

**Figure 13:**
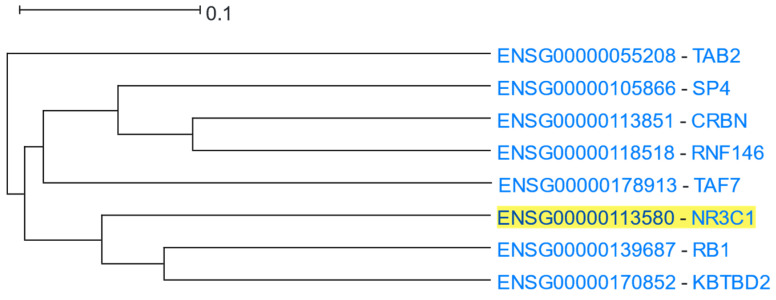
HGCA2.0 *NR3C1* 3 internal node coexpression clade.

NR3C1 (Nuclear Receptor Subfamily 3 Group C Member 1, also known as Glucocorticoid Receptor) is a nuclear receptor of the superfamily of ligand-dependent transcription factors, mediating the physiologic pleiotropic actions of glucocorticoids [106]. NR3C1 is ubiquitously expressed across almost all cell types, during all developmental stages. In the absence of glucocorticoids, the inactive NR3C1 is primarily located in the cytoplasm as a component of a multiprotein complex, including chaperones (of HSP70 and HSP90 family of proteins and PTGES3) and immunophilins (FKBP5 and FKBP4) [107]. Upon ligand binding, NR3C1 is conformationally changed, dissociates from the other proteins of the complex, homodimerises, and translocates into the nucleus, where NR3C1 homodimers bind to glucocorticoid receptor elements (GREs), regulating the expression of target genes [108], resulting in the regulation of up to 10–20% of the human genome [109]. *NR3C1* was used as the driver gene in a HGCA2.0 analysis and the resulting clade was reduced to 3 internal nodes (Figure 13). The most correlated genes with *NR3C1* were *RB1* (RB transcriptional Corepressor 1) and *KBTBD2* (Kelch Repeat and BTB Domain Containing 2). *RB1* encodes for a negative regulator of the cell cycle (G1/S transition) and is known as the first reported oncosuppressor gene [110]. It has been proposed that NR3C1-mediated cell cycle arrest is induced when the activated NR3C1 inhibits the expression of G1-acting cyclin/CDK complexes, leading to Rb hypophosphorylation [111]. Therefore, *RB1* seems to be involved in the NR3C1-mediated cell cycle arrest, and thus, was shown as a closely correlated gene. The function of *KBTBD2* appears to be largely unexplored. KBTBD2 induces PIK3R1 ubiquitination, thus, its proteasome-mediated degradation. In the absence of KBTBD2, the concentration of PIK3R1 increases dramatically [112]. It has been proposed that free PIK3R1 negatively regulates PI3K signalling by competition with the Class IA PI3K complex (which is a heterodimer of PIK3R1 and PIK3CA, PIK3CB or PIK3CD) for binding to phosphotyrosine docking sites [113]. NR3C1 contains two such PI3K recruitment motifs that contribute to the NR3C1-PI3K interaction. The physical interaction between NR3C1 and the PIK3R1 subunit of PI3K is essential for the rapid non-genomic effects of glucocorticoids [114]. In another line of evidence, EZR phosphorylation by SRC induces the association of EZR with KBTBD2 [115]. As SRC is a component of both the plasma membrane and cytoplasmic NR3C1 complexes, mediating non-genomic NR3C1 signalling [108], and EZR is a cross-linker of plasma membrane proteins with actin cytoskeleton [116], EZR-KBTBD2 interaction might contribute to the regulation of non-genomic NR3C1 signalling [117]. Thus, *KBTBD2* may be mechanistically associated with NR3C1 signalling (especially non-genomic signalling).

An HGCA2.0 enrichment analysis showed “intracellular steroid hormone receptor signaling” among the most significantly over-represented GO Biological Process terms (Table 12). The most highly enriched GO Molecular Function terms were: “transcription coactivator activity”, “sequence-specific DNA binding”, “transcription coregulator activity”, “modification-dependent protein binding”, and “nuclear hormone receptor binding”, all of which are congruent with the NR3C1 signalling pathway [118]. Among the most highly enriched GO cellular compartments were the nucleus and nucleoplasm. Glucocorticoid-activated NR3C1 shows heterogeneous organisation in the nucleus, being distributed between the nucleoplasm and membraneless compartments, the so-called nuclear foci; however, the functional significance of this localisation remains elusive [119]. Using the Chromosome Band analysis, both *NR3C1* and *TAF7* were shown as being located in the same cytogenetic region (5q31.3). *NR3C1* and *TAF7* are indeed positioned in relatively close physical proximity (~2Mbps) within 5q31.3; however, it is unlikely that this co-localisation is responsible for their coexpression relationship, as a dozen other genes, that do not appear in the coexpression clade of *NR3C1*, are located between them.

#### 3.1.11. ALS and LGMD Related Genes

Amyotrophic Lateral Sclerosis (ALS) and Limb-Girdle Muscular Dystrophies (LGMD) are neuromuscular conditions with the common characteristic that any one of a number of single gene mutations may cause them.

ALS is characterised by the loss of both upper and lower motor neurons, and is the most common form of motor neuron disorder [120]. Its onset may occur at any age, but peaks considerably among 54–67 years old, initially involving muscle atrophy, which progresses to swallowing difficulties, paralysis, and ultimately to death by neuromuscular respiratory failure. Patients typically survive for 2–5 years after the first symptoms occur, with 5–10% surviving more than 10 years [121]. Variants in some 30 genes are recognised as monogenic causes of ALS [122,123,124] and the disease has high estimated rates of inheritance [125], but for the large majority of patients, a genetic cause has not yet been identified [126]. Some of the known causal genes have functional relationships to one another and can be grouped accordingly by function, but no common functional pathway has been identified and the functions in which they are involved represent a diverse set of cellular processes [127].

LGMD are characterised by progressive atrophy and weakness of the hip and shoulder (limb-girdle) muscles, which may progress to other muscles of the body [128]. Age at onset, severity, and progression of symptoms may vary greatly from case to case [129]. The condition represents a set of genetic disorders with more than 30 different sub-types, most of which are associated with genetic defects in one or several specific known genes [130]. Most of these genes have known functional relationships to several of the others, and three broad categories of cellular function have been recently proposed [131].

To explore gene coexpression functional groupings, each of the causal genes of ALS (Table S1) and LGMD (Table S2) collected through the bibliography and Orphanet [132], were submitted to HGCA2.0, and coexpression clades for each one of them were produced. Clades were then explored manually, with the addition or subtraction of internal nodes, to identify significant functional enrichments.

Several ALS causal genes were found to inhabit gene clades which produce low *p*-value enrichments of terms related to the known functions of the gene (Table S3). These genes include the neuronal nicotinic acetylcholine receptor subunit *CHRNA3* (Cholinergic Receptor Nicotinic Alpha 3 Subunit), whose clade was enriched for related functional terms such as response to nicotine, neuromuscular synaptic transmission, and acetyl choline binding; the kinesin axonal transporter of neurofilament proteins *KIF5A* (Kinesin Family Member 5A), whose clade was enriched for synapse, neuron projection, and nervous system; *UNC13A* (Unc-13 Homolog A) involved in vesicle maturation during exocytosis occupied a clade enriched for SNARE binding, synaptic vesicle cycle, and neurotransmitter secretion; and the *NEFH* (Neurofilament Heavy Chain) clade was enriched for the synapse and spontaneous neurotransmitter secretion, as well as Ras GTPase binding. *MOBP* (myelin-associated oligodendrocyte basic protein), thought to be involved in stabilisation of the myelin sheath, occupied a clade enriched for astrocyte projection, spinal cord injury, and optic disc oedema. Interestingly, *OPTN* (Optineurin), the protein product of which links MYO6 (myosin VI) to the Golgi complex [133], was observed to be coexpressed with a large cluster of genes highly enriched for muscle contraction and actin-myosin filament sliding. None of the ALS causal genes were observed to closely inhabit the same clade.

Similarly to ALS, a number of LGMD causal genes were found to inhabit clades enriched for terms related to the known functions of the gene (Table S4). However, unlike ALS, several clades were identified to include more than one of the disease query genes. *SGCG*, *POMGNT1*, *DES*, *ANO5*, *MYOT*, *SGCD*, *BVES*, and *SGCA* co-occupied a clade enriched for terms such as myofibril, contractile fibre, and muscle structure development. Distinct from this clade, but with enrichment of overlapping and closely related functions, such as myofibril assembly and sarcomere, was a clade co-occupied by *TCAP*, *TTN*, and *CAV3*. Three collagen genes, *COL6A1*, *COL6A2*, and *COL6A3*, were unsurprisingly coexpressed within a clade of extracellular matrix genes. *LIMS2*, a gene encoding for the focal adhesion protein PINCH-2, was coexpressed with a separate clade of genes involved in muscle contraction. *DAG1* (Dystroglycan 1) was coexpressed among other genes that contribute to the dystrophin-associated glycoprotein complex, enriched in that specific term and also the more general term “peripheral nervous system development”. *LMNA*, encoding for part of the nuclear envelope, inhabited a clade that was enriched for cell adhesion and regulation of cellular component movement, as well as integrin binding and focal adhesion.

#### 3.1.12. Growth Hormones

**Figure 14:**
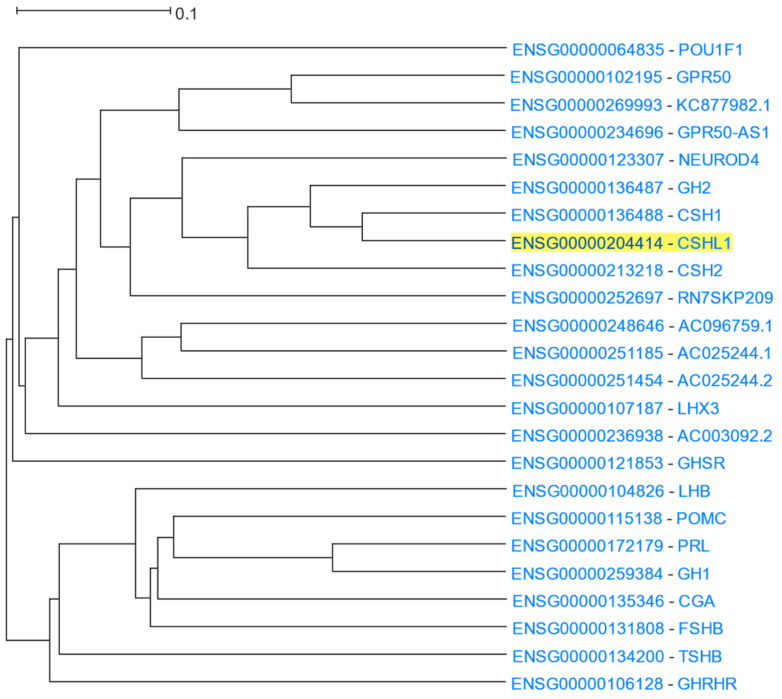
HGCA2.0 *CSHL1* 12 internal node coexpression clade.

The Growth Hormone (GH) family is a cluster of similar genes that encode for proteins related to growth control, whose main member is growth hormone, also known as somatotropin, which is produced in the anterior pituitary gland and has an important role in controlling growth and cell division [134,135]. Specifically, in mammals, growth hormone 1 is encoded by *GH1* [134]. The GH gene family also consists of *GH2* which encodes for placental growth hormone [134], the chorionic somatomammotropin genes (*CSH1* and *CSH2*), and chorionic somatomammotropin-like 1 (*CSHL1*), which are expressed in the placenta. Another member of the growth hormone family is prolactin (*PRL*), which is associated with gland differentiation and lactation in mammals [136,137]. *CSHL1* was used as the input in HGCA2.0 and the resulting coexpression clade included all members of the GH family among the 24 genes listed (Figure 14). A GO Biological Process analysis ranked the response to growth hormone as the top term (Table 13). Enriched terms of growth hormone receptor signalling pathway and regulation of growth also emerged. Moreover, GO Molecular Function and KEGG pathway analyses showed hormone activity and neuroactive ligand-receptor interaction in humans as top functions, respectively, consistent with the presence of a variety of growth hormones and their receptors in the coexpression clade. In addition, DisGeNET and WikiPathways analyses highlighted pituitary diseases and Prader-Willi and Angelman syndromes, in which growth hormone production is known to be affected and therefore lead to developmental delay [138,139]. Finally, a Chromosome Band analysis showed an enrichment of genes in the chromosomal region 17q23.3, evidenced by the fact that five genes of the GH family (*GH1*, *GH2*, *CSH1*, *CSH2*, *CSHL1*) co-localize at the growth hormone locus [134,140].

#### 3.1.13. Antisense Genes

**Figure 15:**
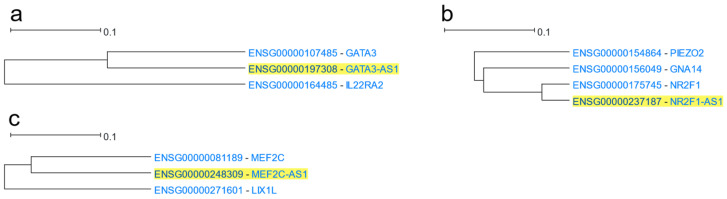
HGCA2.0 coexpression clades of sense genes and their respective antisenses; (**a**) HGCA2.0 *GATA3* 2 internal node coexpression clade; (**b**) HGCA2.0 *NR2F1* 3 internal node coexpression clade; (**c**) HGCA2.0 *MEF2C* 2 internal node coexpression clade.

An antisense gene of a coding or non-coding (sense) gene is transcribed from the opposite strand to the strand the sense transcript is transcribed from. Antisense genes are primarily involved in gene expression regulation, although they might fulfil various roles [141]. HGCA2.0 studies 3627 genes which are labelled as “antisense” in their gene description, 1376 of which include the “-AS” suffix in their HGNC gene symbol. *GATA3*, *NR2F1*, and *MEF2C*, which are genes with known antisense transcripts, were used as drivers in HGCA2.0. In all three cases, the most coexpressed gene to each driver gene was its corresponding antisense transcript, being situated in the adjacent branch (Figure 15), which in turn discloses a possible functional association. Indeed, *GATA3-AS1*-driven tumour growth and metastasis in liver cancer are mediated by GATA3 [142], and *NR2F1-AS1* was shown to have adverse effects in pancreatic cancer by activating the NR2F1/AKT/MTOR axis [143]. In the human genome, the primary transcripts of *NR2F1* and *MEF2C* constitute divergent overlapping pairs with their antisense transcripts, while the primary transcript of *GATA3* and its antisense form a divergent non-overlapping gene pair, with an intergenic distance of less than 1.5 Kbps between their 5′ ends (Figure S8).

### 3.2. Coexpression Tool Benchmarking

13 genes of the use cases described earlier (*C1orf68*, *CSHL1, HLA-DMA, HSP90AA1*, *HSP90B1*, *MT1M*, *NLRC5*, *NR3C1*, *NRP1*, *OR1D2, RPL11*, *STAT1*, and *TMPRSS2*) were used for benchmarking of HGCA2.0 and four other popular webtools (Table S5). The tools’ performances were evaluated based on their produced PPI network metrics, as well as the relevance of their enriched biological terms and their corresponding adjusted *p*-values. For *C1orf68*, HGCA2.0, COXPRESdb, and GTEx-based GeneFriends performed best, while SRA-based GeneFriends did not result in a connected STRING network and ARCHS^4^ produced no coexpression results. For *CSHL1*, HGCA2.0 exhibited the best performance regarding network metrics. Most webtools produced enriched terms related to growth hormone activity. For *HLA-DMA* and *NLRC5*, all webtools except for SRA-based GTEx had dense networks, with HGCA2.0 performing best, showing enriched biological terms related to defence response and antigen processing. For the *HPS90AA1* and *HSP90B1* heat shock protein genes, all webtools, except for SEEK, performed comparably well, showing “Protein Folding” and “Response to endoplasmic reticulum stress” as enriched terms, respectively. For *MT1M*, SRA-based GeneFriends and ARCHS^4^ produced networks with 1 edge each, while the other webtools found no connections. COXPRESdb produced the lowest enrichment *p*-values, while HGCA2.0 produced the second highest ones. For *NR3C1*, SRA-based GeneFriends and ARCHS^4^ performed best regarding network metrics, while HGCA2.0 showed the sparsest network. In addition, HGCA2.0 discovered no enriched terms, while the enriched biological terms discovered by the other webtools were very generic. In the case of *NRP1*, HGCA2.0 and SEEK performed best, both in network metrics as well as enrichment *p*-values. For *RPL11*, all webtools exhibited dense networks with high levels of statistical confidence, although SEEK and SRA-based GeneFriends performed slightly worse. For *OR1D2*, all networks were sparse, with only HGCA2.0, ARCHS^4^ and, to some extent, COXPRESdb producing statistically significant enriched terms related to olfactory receptor biological functions, with HGCA2.0 having by far the lowest *p*-values. GTEx-based GeneFriends had the densest network, but its coexpressed genes were enriched for “sexual reproduction”, a term unrelated to olfactory receptors. It should be mentioned that the analysis of ARCHS^4^ used only 76 genes mapped by STRING, as the webtool outputs a list of a maximum of 100 coexpressed genes. For *STAT1*, HGCA2.0 and COXPRESdb performed best. HGCA2.0 also had the lowest GO Biological Process enrichment term *p*-values. For *TMPRSS2*, COXPRESdb performed best in both network metrics and enrichment *p*-values, while HGCA2.0 had the fourth best performance.

## 4. Discussion

### 4.1. Comparison with Previous HGCA Version

HGCA2.0 has been developed as an upgrade to the original Human Gene Correlation Analysis (HGCA1.0) tool [12], created over 10 years ago. The initial HGCA version included expression data derived from 1959 healthy high-quality Affymetrix Human Genome U133 Plus 2.0 Array Chip samples, which were manually selected as tissue representatives of 4452 high-quality healthy samples in a way to minimise tissue bias. Microarray samples were then normalised using MAS5.0 with default Affymetrix CDF. Since default CDF does not guarantee a one-to-one gene-probe set correspondence, users were required to select one of the available probe sets for their gene of interest and, as the original HGCA was microarray-based, the searchable gene list was also limited, compared to the current knowledge and understanding of the human genome. HGCA1.0 could produce both lists of the most coexpressed genes to the gene of interest or neighbour-joining-based [144] coexpression clades and offered various enrichment analysis categories.

The updated HGCA version is based on 3500 GTEx bulk RNA-Seq samples, which were automatically selected as representatives of the original 16,704 non-cell line ones. GTEx guarantees high-quality samples and healthy tissue conditions, as well as optimal RNA-Seq execution and data preprocessing [11]. In addition, RNA-Seq is a method that is more accurate and sensitive in measuring gene expression in tissue, compared to microarrays. Furthermore, as it is not dependent on probe hybridisation, expressions are not limited to a set of genes. Finally, a UPGMA hierarchical clustering method was used as an alternative to neighbour joining, as its cophenetic distances better corresponded to the original pairwise distances.

In large-scale coexpression analyses which depend on the processing of raw data from different studies, batch correction may be necessary, unless advanced normalisation algorithms, such as SCAN [145], are employed, as in the case with ACT [2,33]. Although HGCA1.0 was based on data from more than 300 studies, which were normalised by MAS5.0, a basic single-channel array normalisation method, no batch correction was applied. As GTEx is a single study, there was no need for batch correction in HGCA2.0. In addition, read counts were normalised using the qsmooth algorithm, which performs best for datasets of various tissues, as is the case of GTEx [27]. HGCA2.0 further contains new and updated biological term libraries for improved enrichment analyses. In HGCA1.0, transcription factor analysis was based on predicted transcription factor binding sites from MATCH [146] hits of Position Weight Matrices from TransFac [147]. On the other hand, experimentally validated gene-transcription factor interactions from ENCODE and ReMap are a unique feature of HGCA2.0, thus, being novel in highlighting the transcription factors which may act as master co-regulators which drive gene coexpression.

### 4.2. Comparison of Coexpression Webtools

To compare the performance of the 5 coexpression webtools, their outputs for 13 driver genes were used for the construction of STRING PPI networks, which served as an independent measure. Since ribosomal proteins are ubiquitously and concurrently expressed for ribosome assembly, coexpression webtools expectedly discovered most ribosomal proteins as coexpressed, resulting in high STRING PPI network metrics and comparable biological term enrichment *p*-values (Table S5). Small differences in such low *p*-values between tools should not be considered significant, as they might depend on even a single coexpressed gene difference. STAT1 is a transcription factor related to defence response genes and all webtools produced enrichments of relevant biological terms, but with highly varying significance levels, with HGCA2.0 having the lowest *p*-value, followed by COXPRESdb, while the two GeneFriends versions were lower than the rest. *NRP1* coexpressed gene lists produced various enrichments depending on the coexpression webtool used, with only HGCA2.0 highlighting the gene’s role in vasculature development. STRING analyses of *NR3C1*, *TMPRSS2*, *MT1M*, and *OR1D2* did not produce dense PPI networks. As a result, webtools that discovered even one more edge than the rest of the tools, exhibited better network metrics. Thus, in those four genes, enrichment analyses were mostly used to determine the best performance. In the cases of *C1orf68*, *CSHL1*, and *NLRC5*, HGCA2.0 performed best. For *HSP90AA1* and *HSP90B1*, COXPRESdb outperformed all other tools, while for *HLA-DOA*, HGCA2.0 performed equally well with COXPRESdb. The performance of coexpression webtools shows variation on a case-to-case basis, possibly attributable to their different ways of calculating coexpression between genes and their different transcriptomic datasets. Overall, COXPRESdb and HGCA2.0 performed best, followed by GTEx-based GeneFriends, SEEK and ARCHS^4^ and, finally, SRA-based GeneFriends. In addition, GTEx-based GeneFriends outperformed its SRA-based version in almost all examples, hinting that the choice of GTEx data by HGCA2.0 is favourable for studying condition-independent gene coexpression. Interestingly, HGCA2.0 and GTEx-based GeneFriends version showed significant differences in performance for specific genes, even though both were based on data from the same source. This may be due to the fact that GeneFriends used all available GTEx samples, without the prior representative sample selection that HGCA2.0 applied, possibly leading to the introduction of tissue biases: The complete GTEx dataset displays great variability in the number of samples per tissue, distorting the depiction of the global coexpression landscape. Furthermore, this effect could be accentuated depending on the selected normalisation process of raw data: GeneFriends GTEx samples were not normalised using a tissue-aware normalisation method, as opposed to the qsmooth algorithm that was used by HGCA2.0. These issues could explain the high performance of GeneFriends in ubiquitously expressed genes (e.g., *RPL11*), and its low performance in stimulus-related (e.g., *STAT1*) or cell type-specific (e.g., *NRP1*) genes.

Apart from each tool’s performance, there are also differences in the presentation of coexpression. All webtools except for HGCA2.0 produce a list of the most coexpressed genes as their main output. While gene lists offer a simple depiction of gene coexpression, they do not constitute a systems biology approach, as they do not show the interconnections between coexpressed genes. SEEK additionally shows a heatmap depicting the expression levels of 100 selected genes from the ordered coexpression gene list in 50 selected datasets, which is a limited approach, as it is restricted to a specific number of genes and samples each time. Coexpressed genes can be visualised as Gene Coexpression Networks (GCNs) in COXPRESdb and GeneFriends, or as UMAP [148] plots in COXPRESdb and ARCHS^4^. GeneFriends GCN is interactive as the user can alter the number of coexpressed genes and the r-value cut-off, while COXPRESdb GCN has a fixed cut-off that does not allow the user to estimate the strength of correlations between the coexpressed genes themselves. The coexpression clade visualisation of HGCA2.0 is easily understood by molecular biologists, who are accustomed to the same depiction in phylogenetic trees. Furthermore, the size of coexpression clades in HGCA2.0 can be altered. Finally, all other webtools, except for SEEK and HGCA2.0, depend on external tools to perform enrichment analysis. External enrichment analysis tools do not replace HGCA2.0’s own enrichment analysis since many of them do not include non-coding RNAs, as in the case of STRING’s enrichment statistics.

### 4.3. Limitations

HGCA2.0 is based on the latest GTEx Release (V8), which uses the GENCODE v26 annotation of the GRCh38 human reference genome assembly. As GENCODE v26 was released on 14 March 2017, genes that were added in later versions of GENCODE were not included in GTEx V8. Likewise, GTEx V8 contains genes of GENCODE v26 which have been rendered obsolete in later versions of GENCODE. As GTEx RNA-Seq FASTQ files are not publicly available, it would be preferable if these data were reprocessed in new GTEx releases using the latest GENCODE version. That would enable HGCA2.0 to work to its full potential. This limitation was encountered in an attempt to study hominin encephalisation using HGCA2.0, where *ARHGAP11B* was selected as the ideal driver gene, as it derived from partial duplication of *ARHGAP11A* after humans and chimpanzees split [149], promotes basal progenitor amplification and neocortex expansion [150], and its deletion may cause microcephaly [151]. However, as *ARHGAP11B* was first introduced in GENCODE v28, it was not included in GTEx V8, thus HGCA2.0 was not able to study its coexpression.

Due to the inherent attributes of the coexpression tree depiction used by HGCA2.0, it is not easy for the user to determine the optimal coexpression clade size for a gene of interest. Selection of the best size may be determined through achieving the lowest possible adjusted *p*-values of enriched terms, by the presence of known gene partners or the topology of the tree itself. Another feature related to tree depiction, is the fact that multiple gene queries are not allowed in HGCA2.0. Furthermore, gene coexpression trees are not able to efficiently portray anti-correlated genes. As gene correlations are converted to non-negative distance values prior to hierarchical clustering, coexpressed genes grouped close to each other usually represent gene partners, but long distances between genes in the coexpression tree do not necessarily relate to negative correlations.

The rationale for selecting olfactory receptor genes as a use case, was that due to their monogenic and monoallelic expression, they would be expected to be fully anti-correlated among themselves (i.e., having Pearson correlation coefficients close to −1). Nevertheless, in HGCA2.0, their pairwise correlations appear close to 0 (i.e., not correlated). The unique olfactory receptor gene coexpression pattern would only be revealed using single-cell RNA-Seq (scRNA-Seq) data, instead of bulk RNA-Seq ones which produce averages of gene expressions due to the nature of tissue sampling, i.e., using parts of the olfactory epithelium which contain multiple olfactory cells. This could explain why olfactory receptor genes were grouped by UPGMA hierarchical clustering on distinct subclades of HGCA2.0. Interestingly, even though STRING v11.5 aims to connect functionally related genes, it failed to correlate olfactory receptor genes, while HGCA2.0 achieved their grouping, even though their correlation values would not sufficiently lead to that conclusion.

Enrichment analysis depends on the annotation quality of each source database. Large parts of the human genome are not properly annotated, if at all, and there are many variations in gene annotations between different databases [152]. Additionally, databases which are based on text evidence may contain misannotated data which may impact the quality of subsequent enrichment analyses. For instance, due to erratic text-mining, DisGeNet falsely linked metallothioneins with metatarsalgia [153] and melatonin deficiency [154], since it misidentified MT1 (type 1 family of the metallothionein superfamily), as MT-I (first metatarsal bone) and MT1 (Melatonin Receptor 1A).

### 4.4. Interpretation of HGCA2.0 Predictions

The prediction potential of HGCA2.0 can be assessed by comparing its output to the existing literature. The use cases demonstrated that HGCA2.0 does indeed have the ability to reproduce known biology. Thus, the gene coexpression clades identified by HGCA 2.0 have the potential to reveal novel mechanistic relationships for human genes, which may give useful insights into cellular processes that involve multiple genes with diverse functional roles. HGCA2.0 analysis is exploratory with no pre-defined significance thresholds, the intention being to show the potential for HGCA2.0 to identify novel gene groupings that may be worthy of future investigation due to their sharing of molecular functions and potential relevance to the understanding of cellular pathology. So, gene coexpression functional relationships were explored in two neuromuscular conditions: ALS and LGMD. Co-occupancy of gene coexpression clades was observed for many of the genes that harbour causal mutations for LGMD, but no ALS causal genes were observed to occupy the same clades as one another. This may reflect the fewer functional groupings that have been proposed for LGMD causal genes [131] compared to ALS causal genes, for which common functional grouping remains a largely unmet challenge [127]. Complete molecular mechanistic explanations of these pathologies, tracing the emergence of a single definable disease (albeit with subtypes and clinical variation) from diverse genetic mutations, remain lacking for both ALS and LGMD.

Constitutively expressed genes would be expected to be correlated amongst themselves in healthy samples, regardless of their differences in biological functions. However, in HGCA2.0, HSP90B1 which is continuously expressed in cytosol, is coexpressed with its functional partners. This would imply that there is regulation of the expression even of constitutively expressed genes, suggesting a revisiting of the term “constitutive” gene.

HGCA2.0 was also tested for its ability to study the coexpression between sense and their antisense genes. Indeed, in three use cases, sense and antisense were next to each other in their respective coexpression clades, with all pairs belonging to the divergent pair class either overlapping or non-overlapping. Divergent genes of the same bidirectional promoter share common proximal regulatory elements which constitute the driving force of their coexpression. The discovery of coexpression between coding and non-coding genes cannot be achieved using microarray-based coexpression webtools or PPI network tools, such as STRING [54] or Genemania [51], as none of them study non-coding RNAs.

## 5. Conclusions

HGCA2.0 is an RNA-Seq-based webtool that performs gene coexpression analysis in *Homo sapiens*. HGCA2.0 has been thoroughly tested for ubiquitously expressed genes, as well as tissue- or condition-specific genes. All use cases were validated by cross-checking the coexpression partners and enrichment results via an extensive bibliographical search. In use cases serving to benchmark HGCA2.0 and other coexpression webtools, using STRING PPI metrics as an independent assessor, HGCA2.0 generally showed the top performance among its competitors. We believe that this new HGCA version will be an important addition to the community of molecular biologists, enabling them to create verifiable hypotheses for gene partnership, especially considering the unique features of HGCA2.0: its user-friendly interface; its biologically relevant output, avoiding information overload; the gene coexpression tree depiction; and the enrichment analysis for verified gene-targeting transcription factors.

## Figures and Tables

**Table 1:** Selected top gene term enrichments of the coexpressed genes to *RPL11* in HGCA2.0.

Category	*p*-Value	Term ID	Description
GO: Biological Process	7.9 × 10^−174^	GO:0006614	SRP-dependent cotranslational protein targeting to membrane
GO: Molecular Function	1.7 × 10^−149^	GO:0003735	Structural constituent of ribosome
GO: Cellular Component	2.0 × 10^−175^	GO:0022626	Cytosolic ribosome
	4.8 × 10^−148^	GO:0044391	Ribosomal subunit
KEGG	4.4 × 10^−133^	hsa03010	Ribosome—*Homo sapiens* (human)
WikiPathways	1.7 × 10^−154^	WP477_r108309	Cytoplasmic Ribosomal Proteins
DisGeNet	4.2 × 10^−47^	C1260899	Anemia, Diamond-Blackfan
Pfam	3.9 × 10^−5^	Ribosomal_L7Ae	Ribosomal protein L7Ae/L30e/S12e/Gadd45 family

**Table 2:** Selected top gene term enrichments of the coexpressed genes to *MT1M* in HGCA2.0.

Category	*p*-Value	Term ID	Description
GO: Biological Process	2.6 × 10^−14^	GO:1990169	Stress response to copper ion
	2.6 × 10^−14^	GO:0010273	Detoxification of copper ion
	2.9 × 10^−14^	GO:0097501	Stress response to metal ion
	2.9 × 10^−14^	GO:0061687	Detoxification of inorganic compound
GO: Molecular Function	8.8 × 10^−6^	GO:0008270	Zinc ion binding
	1.8 × 10^−5^	GO:0046914	Transition metal ion binding
KEGG	1.8 × 10^−11^	hsa04978	Mineral absorption—*Homo sapiens* (human)
WikiPathways	7.9 × 10^−13^	WP3529_r106738	Zinc homeostasis
	3.9 × 10^−12^	WP3286_r106367	Copper homeostasis
Pfam	3.0 × 10^−16^	Metallothio	Metallothionein
ReMap	7.6 × 10^−5^	ZNF879	Zinc finger protein 879
	1.7 × 10^−2^	ZNF26	Zinc finger protein 26

**Table 3:** Selected top gene term enrichments of the coexpressed genes to *HLA-DMA* in HGCA2.0.

Category	*p*-Value	Term ID	Description
GO: Biological Process	1.9 × 10^−19^	GO:0019886	Antigen processing and presentation of exogenous peptide antigen via MHC class II
	1.9 × 10^−19^	GO:0002495	Antigen processing and presentation of peptide antigen via MHC class II
	1.9 × 10^−19^	GO:0002504	Antigen processing and presentation of peptide or polysaccharide antigen via MHC class II
GO: Molecular Function	1.8 × 10^−15^	GO:0023026	MHC class II protein complex binding
	1.2 × 10^−14^	GO:0032395	MHC class II receptor activity
GO: Cellular Component	1.9 × 10^−34^	GO:0042613	MHC class II protein complex
KEGG	2.1 × 10^−24^	hsa04612	Antigen processing and presentation—*Homo sapiens* (human)
Pfam	1.6 × 10^−25^	C1-set	Immunoglobulin C1-set domain
	4.4 × 10^−16^	MHC_II_alpha	Class II histocompatibility antigen, alpha domain
	1.0 × 10^−15^	MHC_II_beta	Class II histocompatibility antigen, beta domain
ReMap	2.8 × 10^−4^	SMAD5	SMAD family member 5
	2.8 × 10^−4^	ZBED1	Zinc finger BED-type containing 1

**Table 4:** Selected top gene term enrichments of the coexpressed genes to *NLRC5* in HGCA2.0.

Category	FDR	Term ID	Description
GO: Biological Process	3.1 × 10^−17^	GO:0042590	Antigen processing and presentation of exogenous peptide antigen via MHC class I
	1.0 × 10^−12^	GO:0019221	Cytokine-mediated signaling pathway
GO: Molecular Function	3.3 × 10^−4^	GO:0042288	MHC class I protein binding
GO: Cellular Component	4.7 × 10^−17^	GO:0042612	MHC class I protein complex
KEGG Pathways	2.2 × 10^−11^	hsa04612	Antigen processing and presentation—*Homo sapiens* (human)
Pfam	2.8 × 10^−13^	MHC_I_C	MHC_I C-terminus
	1.4 × 10^−4^	CARD	Caspase recruitment domain

**Table 5:** Selected top gene term enrichments of the coexpressed genes to *STAT1* in HGCA2.0.

Category	*p*-Value	Term ID	Description
GO: Biological Process	2.2 × 10^−33^	GO:0051607	Defense response to virus
KEGG	7.7 × 10^−11^	hsa05160	Hepatitis C—*Homo sapiens* (human)
	8.4 × 10^−11^	hsa05164	Influenza A—*Homo sapiens* (human)
WikiPathways	2.1 × 10^−11^	WP4880_r109979	Host-pathogen interaction of human corona viruses—Interferon induction
	3.4 × 10^−8^	WP4868_r109974	Type I Interferon Induction and Signaling During SARS-CoV-2 Infection
Pfam	6.0 × 10^−11^	OAS1_C	2’-5’-oligoadenylate synthetase 1, domain 2, C-terminus
DisGeNet	4.7 × 10^−28^	C0021400	Influenza
	6.3 × 10^−13^	C0042769	Virus Diseases
	1.8 × 10^−10^	C0019196	Hepatitis C
ENCODE	2.6 × 10^−40^	STAT2	Signal transducer and activator of transcription 2
ReMap	5.9 × 10^−16^	STAT2	Signal transducer and activator of transcription 2

**Table 6:** Selected top gene term enrichments of the coexpressed genes to *TMPRSS2* in HGCA2.0.

Category	*p*-Value	Term ID	Description
GO: Biological Process	1.7 × 10^−4^	GO:0007043	Cell-cell junction assembly
	3.2 × 10^−4^	GO:0030855	Epithelial cell differentiation
	3.2 × 10^−4^	GO:0045216	Cell-cell junction organization
	9.6 × 10^−4^	GO:0060429	Epithelium development
GO: Cellular Component	1.4 × 10^−3^	GO:0043296	Apical junction complex
	1.4 × 10^−3^	GO:0005911	Cell-cell junction
KEGG	1.5 × 10^−3^	hsa04514	Cell adhesion molecules (CAMs)—*Homo sapiens* (human)
	1.5 × 10^−3^	hsa04530	Tight junction—*Homo sapiens* (human)
Encode	8.9 × 10^−5^	ZNF217	Zinc finger protein 217
	4.2 × 10^−4^	ESR1	Estrogen receptor 1
	4.3 × 10^−3^	ZBTB7A	Zinc finger and BTB domain containing 7A

**Table 7:** Selected top gene term enrichments of the coexpressed genes to *C1orf68* in HGCA2.0.

Category	*p*-Value	Term ID	Description
GO: Biological Process	2.8 × 10^−19^	GO:0031424	Keratinization
	1.6 × 10^−18^	GO:0008544	Epidermis development
WikiPathways	6.9 × 10^−5^	WP2877_r105854	Vitamin D Receptor Pathway
Pfam	2.1 × 10^−31^	LCE	Late cornified envelope
Chromosome Band	7.4 × 10^−29^		1q21.3

**Table 8:** Selected top gene term enrichments of the coexpressed genes to *HSP90AA1* in HGCA2.0.

Category	*p*-Value	Term ID	Description
GO: Biological Process	2.9 × 10^−17^	GO:0006457	Protein folding
	7.7 × 10^−13^	GO:1900034	Regulation of cellular response to heat
	1.1 × 10^−9^	GO:0061077	Chaperone-mediated protein folding
GO: Molecular Function	7.8 × 10^−12^	GO:0051082	Unfolded protein binding
	1.5 × 10^−10^	GO:0051087	Chaperone binding
	3.4 × 10^−10^	GO:0031072	Heat shock protein binding
GO: Cellular Component	3.1 × 10^−12^	GO:0101031	Chaperone complex
ENCODE	1.1 × 10^−21^	HSF1	Heat shock transcription factor 1
	2.6 × 10^−20^	PPARGC1A	PPARG coactivator 1 alpha (PPARGC1A)
DisGeNET	6.4 × 10^−4^	C0949664	Tauopathies
Pfam	3.4 × 10^−9^	CS	CS domain
	2.0 × 10^−5^	HSP90	Hsp90 protein
	1.1 × 10^−4^	HSP70	Hsp70 protein

**Table 9:** Selected top gene term enrichments of the coexpressed genes to *HSP90B1* in HGCA2.0.

Category	*p*-Value	Term ID	Description
GO: Biological Process	2.8 × 10^−14^	GO:0034976	Response to endoplasmic reticulum stress
	1.3 × 10^−10^	GO:0035966	Response to topologically incorrect protein
	6.7 × 10^−10^	GO:0034975	Protein folding in endoplasmic reticulum
GO: Molecular Function	2.4 × 10^−6^	GO:0003756	Protein disulfide isomerase activity
	2.4 × 10^−6^	GO:0016864	Intramolecular oxidoreductase activity, transposing S-S bonds
	3.2 × 10^−6^	GO:0051087	Chaperone binding
	4.6 × 10^−6^	GO:0051082	Unfolded protein binding
GO: Cellular Component	5.8 × 10^−14^	GO:0005788	Endoplasmic reticulum lumen
	2.9 × 10^−10^	GO:0034663	Endoplasmic reticulum chaperone complex
ENCODE	3.1 × 10^−6^	SP2	Sp2 transcription factor
DisGeNET	1.4 × 10^−7^	C1846707	Spinocerebellar Ataxia 17

**Table 10:** Selected top gene term enrichments of the coexpressed genes to *NRP1* in HGCA2.0.

Category	*p*-Value	Term ID	Description
GO: Biological Process	4.6 × 10^−19^	GO:0001944	Vasculature development
	4.6 × 10^−19^	GO:0072358	Cardiovascular system development
	5.5 × 10^−19^	GO:0048514	Blood vessel morphogenesis
GO: Molecular Function	3.1 × 10^−4^	GO:0004714	Transmembrane receptor protein tyrosine kinase activity
	3.1 × 10^−4^	GO:0001605	Adrenomedullin receptor activity
GO: Cellular Component	3.4 × 10^−4^	GO:1903143	Adrenomedullin receptor complex
KEGG Pathways	3.8 × 10^−2^	hsa04514	Cell adhesion molecules (CAMs)—*Homo sapiens* (human)
	3.8 × 10^−2^	hsa05418	Fluid shear stress and atherosclerosis—*Homo sapiens* (human)
	3.8 × 10^−2^	hsa04270	Vascular smooth muscle contraction—*Homo sapiens* (human)
WikiPathways	6.5 × 10^−4^	WP3943_r106492	Robo4 and VEGF Signaling Pathways Crosstalk
	6.5 × 10^−4^	WP3888_r108912	VEGFA-VEGFR2 Signaling Pathway
DisGeNET	2.7 × 10^−8^	C1519670	Tumour Angiogenesis
	1.2 × 10^−7^	C1658953	Tumour vasculature
Pfam	3.9 × 10^−5^	RAMP	Receptor activity modifying family
	3.9 × 10^−5^	CD34_antigen	CD34/Podocalyxin family
	3.9 × 10^−5^	Sox17_18_mid	Sox 17/18 central domain

**Table 11:** Selected top gene term enrichments of the coexpressed genes to *OR1D2* in HGCA2.0.

Category	*p*-Value	Term ID	Description
GO: Biological Process	7.2 × 10^−269^	GO:0050911	Detection of chemical stimulus involved in sensory perception of smell
GO: Molecular Function	1.5 × 10^−285^	GO:0004984	Olfactory receptor activity
	9.1 × 10^−236^	GO:0004930	G protein-coupled receptor activity
GO: Cellular Component	9.3 × 10^−65^	GO:0016021	Integral component of membrane
KEGG	5.9 × 10^−225^	hsa04740	Olfactory transduction—*Homo sapiens* (human)
WikiPathways	1.2 × 10^−18^	WP455_r106426	GPCRs, Class A Rhodopsin-like
	3.8 × 10^−8^	WP4558_r107928	Overview of interferons-mediated signaling pathway
Pfam	1.2 × 10^−278^	7tm_4	Olfactory receptor

**Table 12:** Selected top gene term enrichments of the coexpressed genes to *NR3C1* in HGCA2.0.

Category	*p*-Value	Term ID	Description
GO: Biological Process	9.7 × 10^−4^	GO:0030522	Intracellular steroid hormone receptor signaling pathway
GO: Molecular Function	1.2 × 10^−2^	GO:0003713	Transcription coactivator activity
	1.3 × 10^−2^	GO:0140030	Modification-dependent protein binding
	1.3 × 10^−2^	GO:0035257	Nuclear hormone receptor binding
GO: Cellular Component	1.6 × 10^−2^	GO:0005654	Nucleoplasm
Chromosome Band	4.5 × 10^−4^		5q31.3

**Table 13:** Selected top gene term enrichments of the coexpressed genes to *CSHL1* in HGCA2.0.

Category	*p*-Value	Term ID	Description
GO: Biological Process	1.5 × 10^−12^	GO:0060416	Response to growth hormone
	7.5 × 10^−12^	GO:0060396	Growth hormone receptor signaling pathway
	7.3 × 10^−9^	GO:0040008	Regulation of growth
GO: Molecular Function	6.6 × 10^−19^	GO:0005179	Hormone activity
	7.8 × 10^−13^	GO:0005131	Growth hormone receptor binding
KEGG Pathway	2.3 × 10^−16^	hsa04080	Neuroactive ligand-receptor interaction—*Homo sapiens* (human)
	3.1 × 10^−9^	hsa04935	Growth hormone synthesis, secretion and action—*Homo sapiens* (human)
DisGeNET	2.1 × 10^−15^	C0013338	Pituitary dwarfism
	2.7 × 10^−15^	C0032002	Pituitary Diseases
WikiPathways	3.9 × 10^−5^	WP3998_r106536	Prader-Willi and Angelman Syndrome
Chromosome Band	3.4 × 10^−10^		17q23.3

## Data Availability

HGCA2.0 is available as a web service through https://www.michalopoulos.net/hgca2.0/ (accessed on 20 January 2023); Gene counts were downloaded from https://storage.googleapis.com/gtex_analysis_v8/rna_seq_data/GTEx_Analysis_2017-06-05_v8_RNASeQCv1.1.9_gene_reads.gct.gz (accessed on 20 January 2023); TPMs were downloaded from https://storage.googleapis.com/gtex_analysis_v8/rna_seq_data/GTEx_Analysis_2017-06-05_v8_RNASeQCv1.1.9_gene_tpm.gct.gz (accessed on 20 January 2023); Sample metadata were downloaded from https://storage.googleapis.com/gtex_analysis_v8/annotations/GTEx_Analysis_v8_Annotations_SampleAttributesDS.txt (accessed on 20 January 2023) and https://www.ebi.ac.uk/arrayexpress/files/E-MTAB-5214/E-MTAB-5214.sdrf.txt (accessed on 20 January 2023); ReMap2020 data were downloaded from http://remap.univ-amu.fr/storage/remap2020/hg38/MACS2/remap2020_crm_macs2_hg38_v1_0.bed.gz (accessed on 20 January 2023); ENCODE data were downloaded from https://maayanlab.cloud/static/hdfs/harmonizome/data/encodetfppi/gene_attribute_edges.txt.gz (accessed on 20 January 2023); Cytoband coordinates were downloaded from https://ftp.ncbi.nlm.nih.gov/pub/gdp/ideogram_9606_GCF_000001305.15_400_V1 (accessed on 20 January 2023) https://ftp.ncbi.nlm.nih.gov/pub/gdp/ideogram_9606_GCF_000001305.15_550_V1 (accessed on 20 January 2023) and https://ftp.ncbi.nlm.nih.gov/pub/gdp/ideogram_9606_GCF_000001305.15_850_V1 (accessed on 20 January 2023); Gene Ontology data were downloaded from http://purl.obolibrary.org/obo/go.obo (accessed on 20 January 2023); WikiPathways were downloaded from https://wikipathways-data.wmcloud.org/20200410/gmt/wikipathways-20200410-gmt-Homo_sapiens.gmt (accessed on 20 January 2023); OMIM data were downloaded after registration from https://www.omim.org/downloads (accessed on 20 January 2023); DisGeNet data were downloaded from https://www.disgenet.org/static/disgenet_ap1/files/downloads/all_gene_disease_associations.tsv.gz (accessed on 20 January 2023); Pfam data were downloaded from https://ftp.ebi.ac.uk/pub/databases/Pfam/releases/Pfam33.1/Pfam-A.clans.tsv.gz (accessed on 20 January 2023); Genomic data were downloaded from http://jan2020.archive.ensembl.org/biomart/martview/ (accessed on 20 January 2023); KEGG Pathways were downloaded from https://www.genome.jp/dbget-bin/get_linkdb?-t+pathway+gn:T01001 (accessed on 20 January 2023).

## Supplementary Materials

The following supporting information can be downloaded at: https://www.mdpi.com/article/10.3390/cells12030388/s1, Figure S1: GTEx sample number per tissue before and after automatic pruning (“All Samples” and “Selected Samples”, respectively); Figure S2: Hierarchical clustering of GTEx samples using UPGMA after qsmooth normalisation. Long lines represent the 3500 samples which were retained after automatic pruning and short lines the 13,204 samples that were pruned. Samples are colour-coded by tissue, using the same colours as the ones on the GTEx website. Tissue names are only shown once, closest to where most of their samples are grouped; Figure S3: Genomic coordinates of *CIITA* and *AC133065.3* (appearing as its Ensembl transcript stable ID—ENST00000625054). Figure was adapted from Ensembl genome browser; Figure S4: Genomic location containing LCE genes and *C1orf68*. Figure was adapted from Ensembl genome browser; Figure S5: Multiple sequence alignment of the protein sequences of LCE genes appearing in the HGCA2.0 coexpression clade and *C1orf68* by MUSCLE; Figure S6: Discovery of tandem repeats in C1orf68 protein sequence using HHrepID; Figure S7: HGCA2.0 *OR1D2* 98 internal node coexpression clade; Figure S8: Genomic coordinates of sense genes and their respective antisenses in a 6Kbps window: (a) Genomic region of *GATA3* and *GATA3-AS1*, which constitute a divergent non-overlapping pair; (b) Genomic region of *NR2F1* and *NR2F1-AS1*, which comprise a divergent overlapping pair; (c) Genomic region of *MEF2C* and *MEF2C-AS1*, which form a divergent overlapping pair. Figures were adapted from Ensembl genome browser; Table S1: ALS causal gene lists that were used as individual inputs in HGCA2.0; Table S2: LGMD causal gene lists that were used as individual inputs in HGCA2.0; Table S3: ALS causal genes which produced significant *p*-value enrichments in their HGCA2.0 coexpression clades and their accompanying enrichments; Table S4: LGMD causal genes which produced significant *p*-value enrichments in their HGCA2.0 coexpression clades and their accompanying enrichments; Table S5: STRING metrics for 13 genes in HGCA2.0, COXPRESdb, the SRA and GTEx versions of GeneFriends, SEEK and ARCHS^4^ coexpression webtools.
