# Supplementary material for: HGCA2.0: An RNA-Seq Based Webtool for Gene Coexpression Analysis in Homo sapiens
Source: Cells. 2023 Jan 21;12(3):388. doi: 10.3390/cells12030388 (PMC9913097; doi:10.3390/cells12030388)
Supplement: Supplementary file 1 [file cells-12-00388-s001.zip › Supplementary Figure S3.pdf]

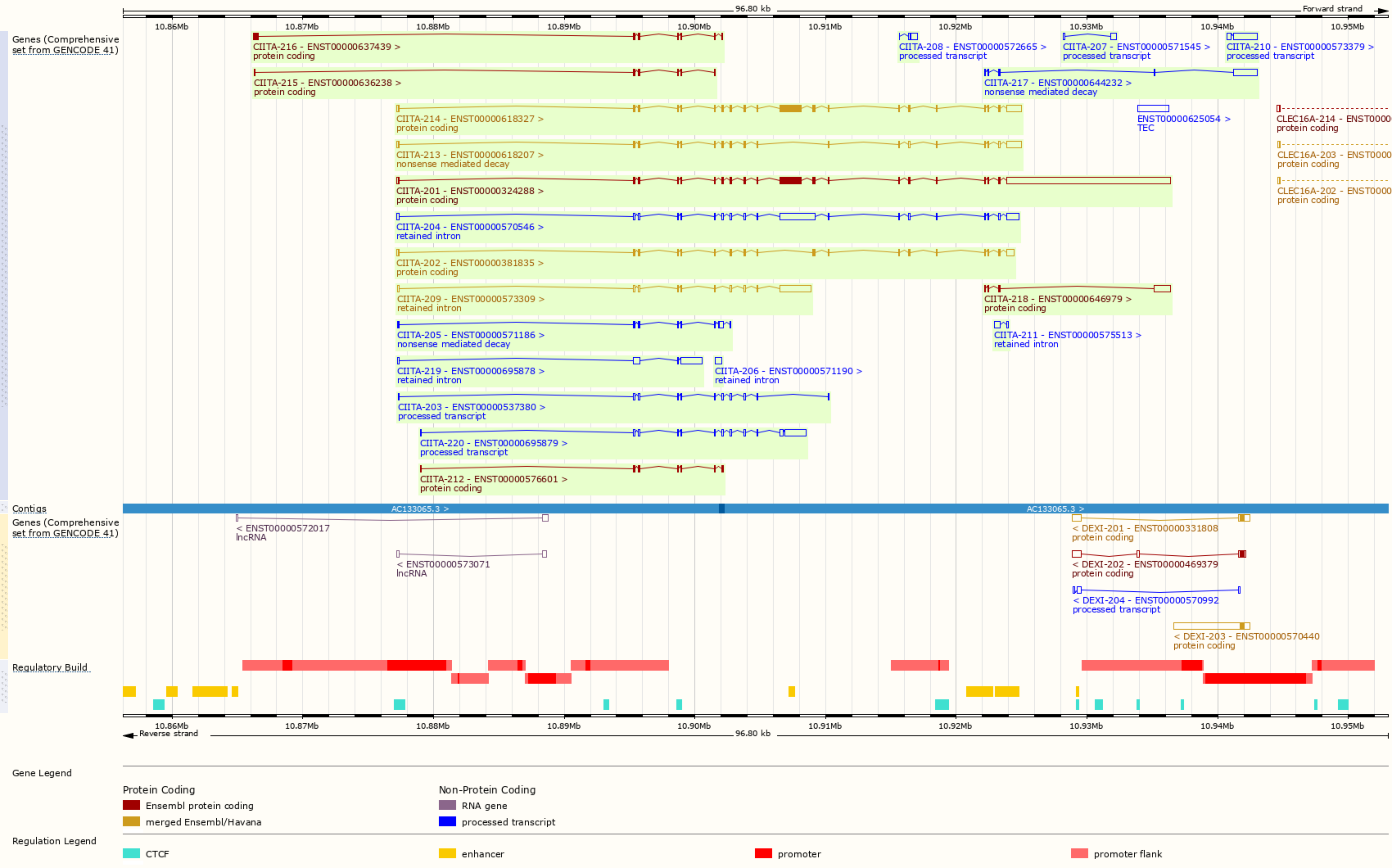
96.80 kb
Forward strand
10.86Mb
10.87Mb
10.88Mb
10.89Mb
10.90Mb
10.91Mb
10.92Mb
10.93Mb
10.94Mb
10.95Mb
Genes (Comprehensive set from GENCODE 41)
CIITA-216 - ENST00000637439 >
protein coding
CIITA-208 - ENST00000572665 >
processed transcript
CIITA-207 - ENST00000571545 >
processed transcript
CIITA-210 - ENST00000573379 >
processed transcript
CIITA-215 - ENST00000636238 >
protein coding
CIITA-217 - ENST00000644232 >
nonsense mediated decay
CIITA-214 - ENST00000618327 >
protein coding
ENST00000625054 >
TEC
CLEC16A-214 - ENST0000
protein coding
CIITA-213 - ENST00000618207 >
nonsense mediated decay
CLEC16A-203 - ENST0000
protein coding
CIITA-201 - ENST00000324288 >
protein coding
CLEC16A-202 - ENST0000
protein coding
CIITA-204 - ENST00000570546 >
retained intron
CIITA-202 - ENST00000381835 >
protein coding
CIITA-209 - ENST00000573309 >
retained intron
CIITA-218 - ENST00000646979 >
protein coding
CIITA-205 - ENST00000571186 >
nonsense mediated decay
CIITA-211 - ENST00000575513 >
retained intron
CIITA-219 - ENST00000695878 >
retained intron
CIITA-206 - ENST00000571190 >
retained intron
CIITA-203 - ENST00000537380 >
processed transcript
CIITA-220 - ENST00000695879 >
processed transcript
CIITA-212 - ENST00000576601 >
protein coding
Contigs
AC133065.3 >
AC133065.3 >
Genes (Comprehensive set from GENCODE 41)
< ENST00000572017
lncRNA
< DEXI-201 - ENST00000331808
protein coding
< ENST00000573071
lncRNA
< DEXI-202 - ENST00000469379
protein coding
< DEXI-204 - ENST00000570992
processed transcript
< DEXI-203 - ENST00000570440
protein coding
Regulatory Build
10.86Mb
10.87Mb
10.88Mb
10.89Mb
10.90Mb
10.91Mb
10.92Mb
10.93Mb
10.94Mb
10.95Mb
Reverse strand
96.80 kb
Gene Legend
Protein Coding
Ensembl protein coding
merged Ensembl/Havana
Non-Protein Coding
RNA gene
processed transcript
Regulation Legend
CTCF
enhancer
promoter
promoter flank
