# Supplementary material for: HGCA2.0: An RNA-Seq Based Webtool for Gene Coexpression Analysis in Homo sapiens
Source: Cells. 2023 Jan 21;12(3):388. doi: 10.3390/cells12030388 (PMC9913097; doi:10.3390/cells12030388)
Supplement: Supplementary file 1 [file cells-12-00388-s001.zip › Supplementary Figure S6.pdf]

ID	Prob	P-val	Loc	Sequence
A1	20.94	2.9e-04	10-17	--FPPSCVKG--.......
A2	25.35	4.9e-02	18-29	SGLGAGQGSNGA.......
A3	71.06	1.4e-10	30-37	--SVKCPVPC--qtqt...
A4	73.22	1.8e-10	42-51	-VCVTGPAPCP-tq.....
A5	75.74	2.5e-11	54-63	-TYVKYQVPCQ-tq.....
A6	73.36	3.3e-12	66-76	-TYVKCPAPCQR.......
A7	80.63	5.4e-17	77-87	-TYVKYPTPCQT.......
A8	76.78	9.7e-15	88-97	--YVKCPAPCQT.......
A9	77.40	2.9e-13	98-106	-TYVKCPTPC--.......
A10	78.08	6.5e-15	107-118	QTYVKCPAPCQM.......
A11	78.53	3.8e-13	119-128	-TYIKSPAPCQ-tqt....
A12	71.08	5.3e-11	132-142	-CYVQGASPCQS.......
A13	62.37	2.4e-07	143-152	-YYVQAPASGS-tsq....
A14	33.01	1.4e-04	156-165	-YCVTDPCSAP-cs.....
A15	44.52	1.3e-04	168-177	-TSYCCLAPRT-fgvsplr
A16	25.31	6.8e-02	185-195	-RWIQRPQNCNT.......
