# Supplementary figures and images for: HGCA2.0: An RNA-Seq Based Webtool for Gene Coexpression Analysis in Homo sapiens
Source: Cells. 2023 Jan 21;12(3):388. doi: 10.3390/cells12030388 (PMC9913097; doi:10.3390/cells12030388)

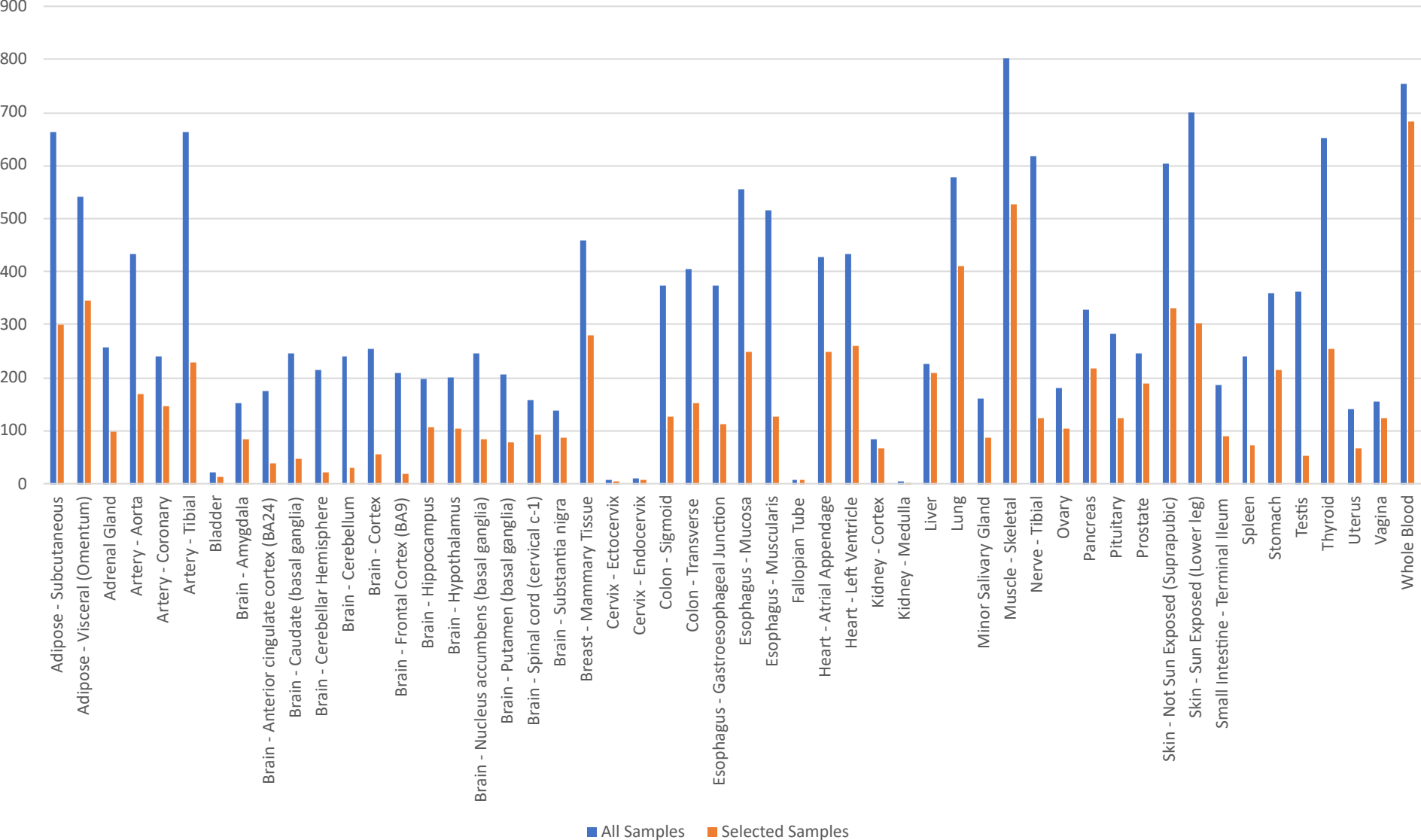

Supplement: Supplementary file 1 [file cells-12-00388-s001.zip › Supplementary Figure S1.pdf]

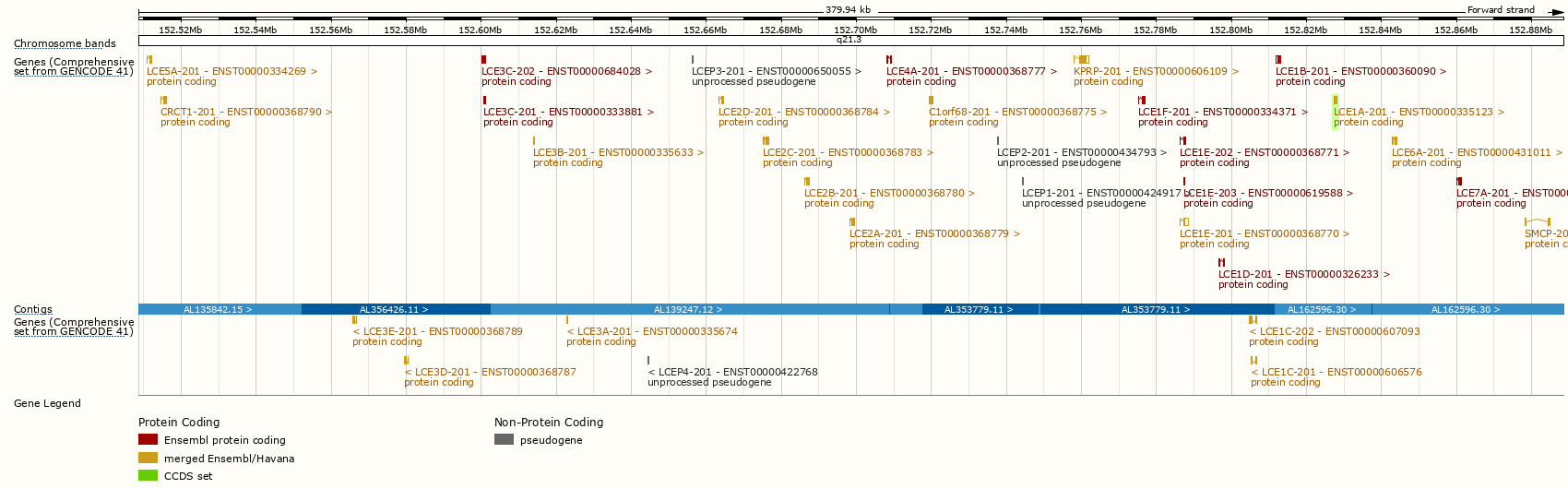

Supplement: Supplementary file 1 [file cells-12-00388-s001.zip › Supplementary Figure S4.png]

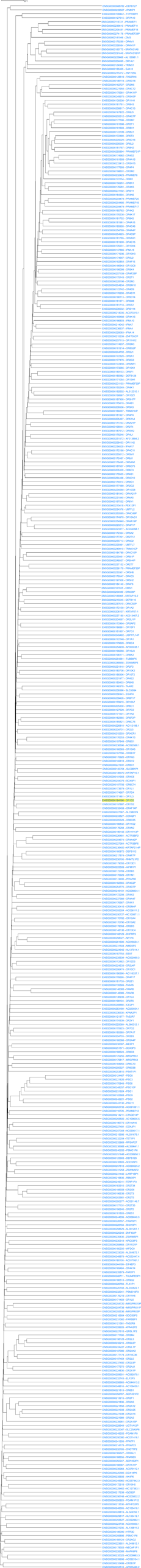

Supplement: Supplementary file 1 [file cells-12-00388-s001.zip › Supplementary Figure S7.pdf]

a

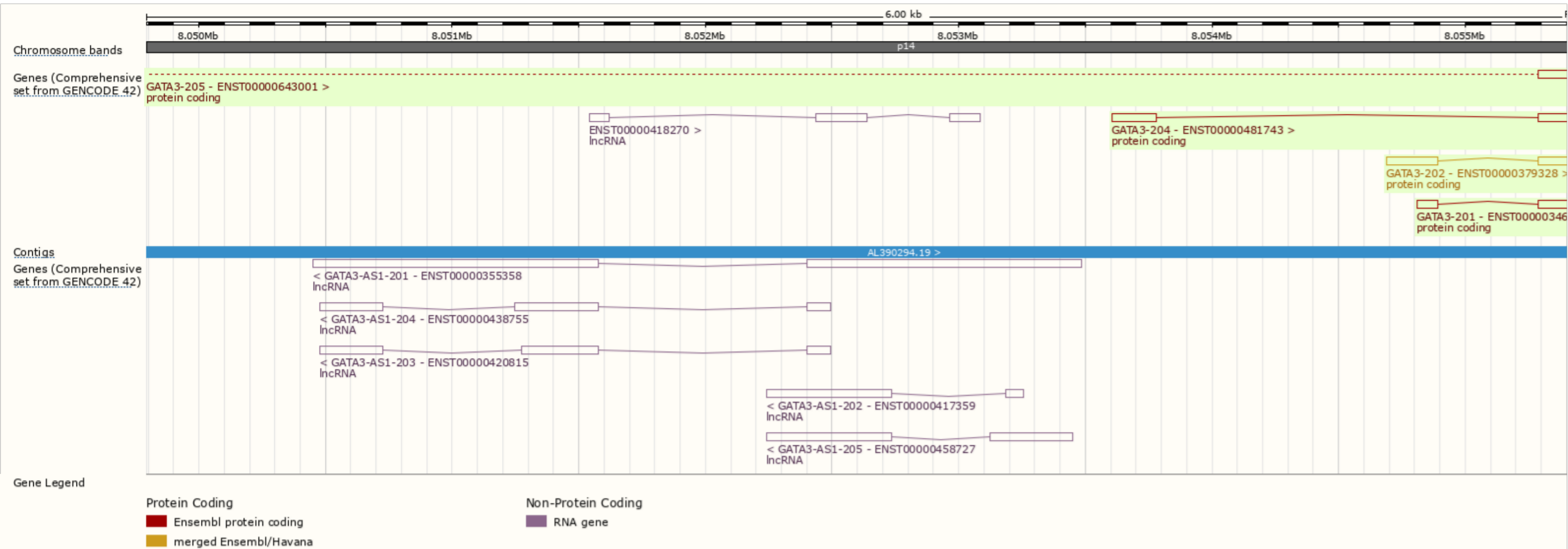

b

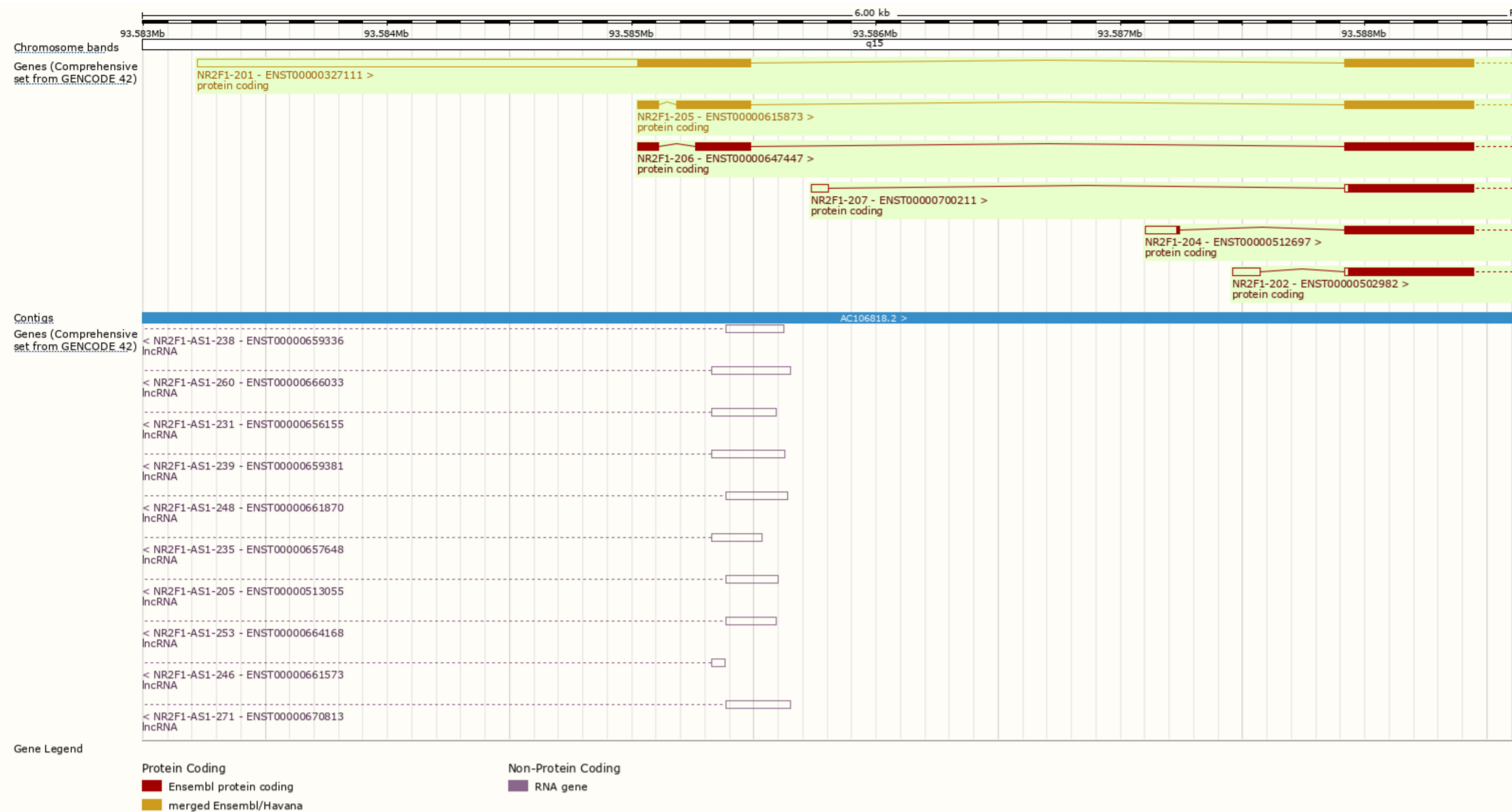

c

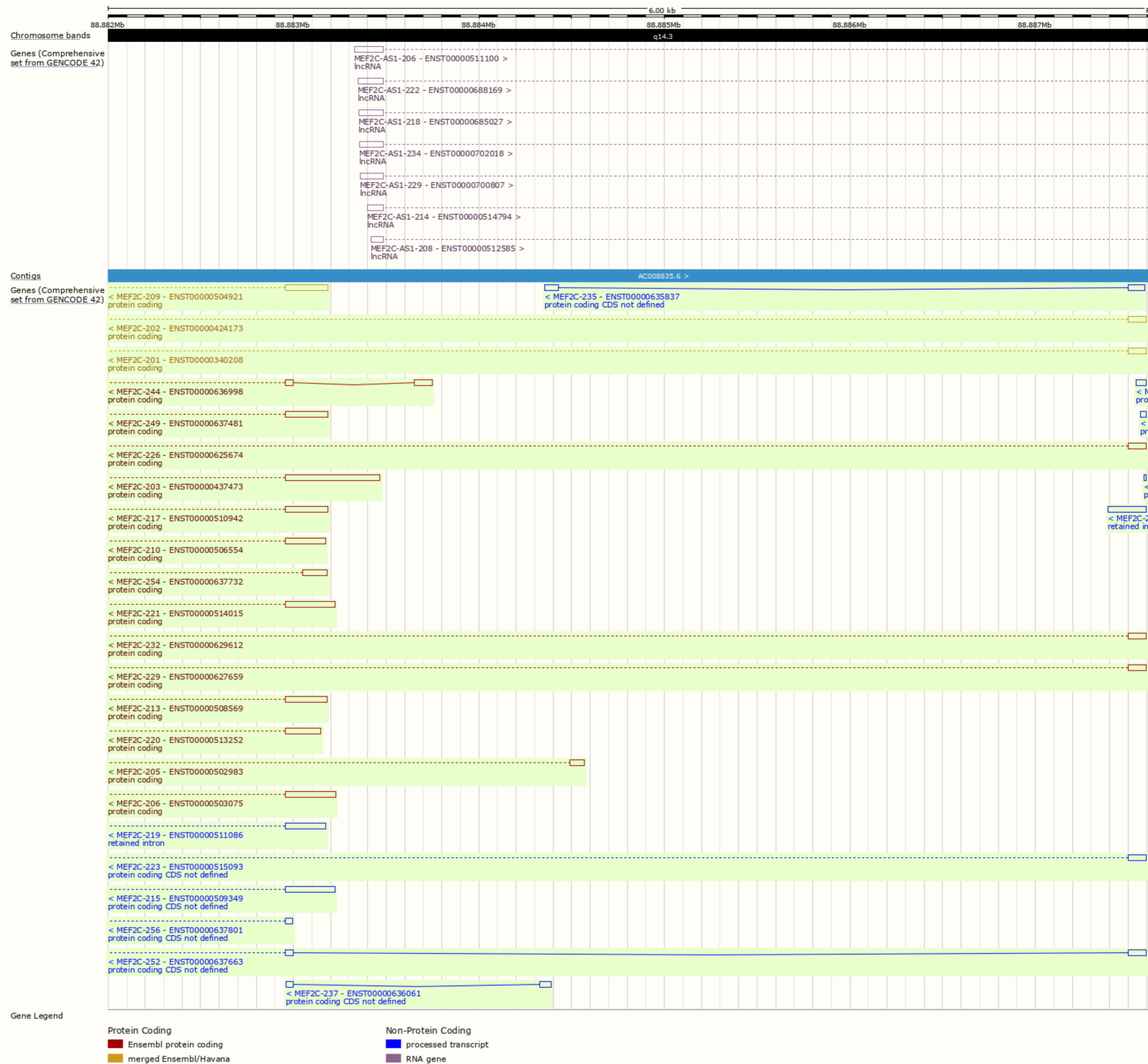

Supplement: Supplementary file 1 [file cells-12-00388-s001.zip › Supplementary Figure S8.pdf]
