# Supplementary material for: HGCA2.0: An RNA-Seq Based Webtool for Gene Coexpression Analysis in Homo sapiens
Source: Cells. 2023 Jan 21;12(3):388. doi: 10.3390/cells12030388 (PMC9913097; doi:10.3390/cells12030388)
Supplement: Supplementary file 1 [file Supplementary Figure S2.pdf › Supplementary Figure S2.pdf]

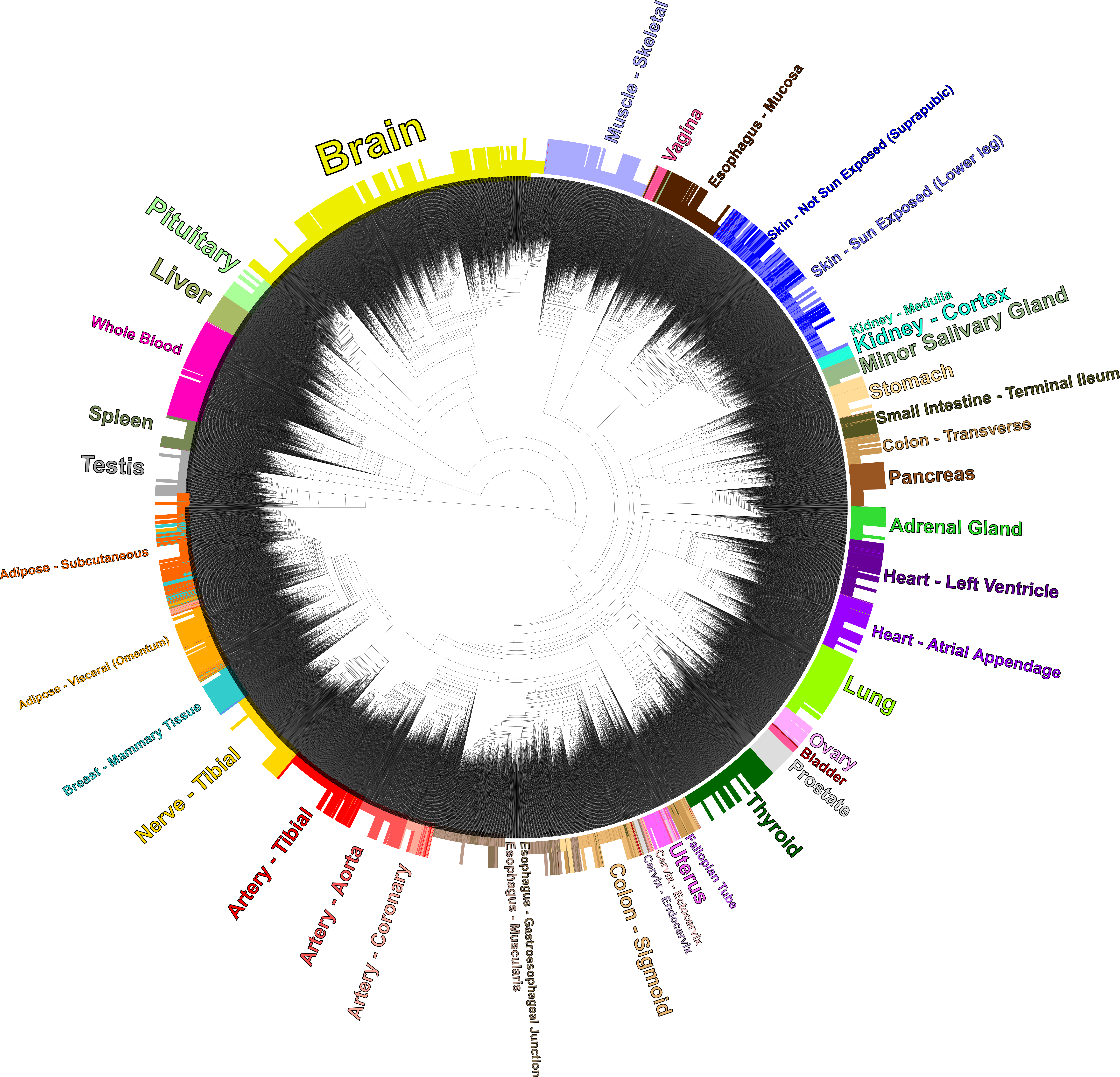
Brain
Muscle - Skeletal
Vagina
Esophagus - Mucosa
Skin - Not Sun Exposed (Suprapubic)
Skin - Sun Exposed (Lower leg)
Kidney - Medulla
Kidney - Cortex
Minor Salivary Gland
Stomach
Small Intestine - Terminal Ileum
Colon - Transverse
Pancreas
Adrenal Gland
Heart - Left Ventricle
Heart - Atrial Appendage
Lung
Ovary
Bladder
Prostate
Thyroid
Fallopian Tube
Uterus
Cervix - Ectocervix
Cervix - Endocervix
Colon - Sigmoid
Esophagus - Gastroesophageal Junction
Esophagus - Muscularis
Artery - Coronary
Artery - Aorta
Artery - Tibial
Nerve - Tibial
Breast - Mammary Tissue
Adipose - Visceral (Omentum)
Adipose - Subcutaneous
Testis
Spleen
Whole Blood
Liver
Pituitary
